# A hepatitis B virus-derived peptide combined with HBsAg exerts an anti-HBV effect in an HBV transgenic mouse model as a therapeutic vaccine

**DOI:** 10.3389/fimmu.2023.1155637

**Published:** 2023-06-02

**Authors:** Yu-Min Choi, Dong Hyun Kim, Junghwa Jang, Bum-Joon Kim

**Affiliations:** ^1^ Department of Microbiology and Immunology, College of Medicine, Seoul National University, Seoul, Republic of Korea; ^2^ Department of Biomedical Sciences, College of Medicine, Seoul National University, Seoul, Republic of Korea; ^3^ Liver Research Institute, College of Medicine, Seoul National University, Seoul, Republic of Korea; ^4^ Cancer Research Institute, College of Medicine, Seoul National University, Seoul, Republic of Korea; ^5^ Department of Microbiology and Immunology, Seoul National University Medical Research Center (SNUMRC), Seoul, Republic of Korea

**Keywords:** hepatitis B virus (HBV), therapeutic vaccination, adjuvant, type 1 interferon (IFN-I), (HBV)-derived peptide, Poly6, DC maturation, cell mediated immune (CMI) response

## Abstract

**Introduction:**

For complete or functional cure of hepatitis B virus (HBV) infection, application of immunotherapy is now being attempted. Recently, we reported that a 6-mer hepatitis B virus (HBV)-derived peptide, Poly6, exerts a strong anticancer effect in tumor-implanted mice through inducible nitric oxide synthase (iNOS)-producing DCs (Tip-DCs) in a type 1 interferon (IFN-I)-dependent manner, suggesting its potential as a vaccine adjuvant.

**Methods:**

In this study, we explored the potential of Poly6 in combination with HBsAg as a therapeutic vaccine against hepatitis B virus infection. We investigated the immunotherapeutic potential of Poly6 combined with HBsAg vaccination against hepatitis B virus infection in C57BL/6 mice or an HBV transgenic mouse model.

**Results:**

In C57BL/6 mice, Poly6 enhanced DC maturation and DC migration capacity in an IFN-I-dependent manner. Moreover, the addition of Poly6 to alum in combination with HBsAg also led to enhanced HBsAg-specific cell-mediated immune (CMI) responses, suggesting its potential as an adjuvant of HBsAg-based vaccines. In HBV transgenic mice, vaccination with Poly6 combined with HBsAg exerted a strong anti-HBV effect via induction of HBV-specific humoral and cell-mediated immune responses. In addition, it also induced HBV-specific effector memory T cells (T_EM_).

**Discussion:**

Our data indicated that vaccination with Poly6 in combination with HBsAg exerts an anti-HBV effect in HBV transgenic mice, which is mainly mediated by HBV-specific CMI and humoral immune responses via IFN-I-dependent DC activation, suggesting the feasibility of Poly6 as an adjuvant for an HBV therapeutic vaccine.

## Introduction

Although preventive vaccines and effective nucleos(t)ide analogs (NAs) are currently available for combating hepatitis B virus (HBV) infection, HBV infections remain a major global health burden, with more than 350 million people chronically infected and approximately 786000 patients dying annually worldwide due to hepatitis B virus (HBV)-related diseases, including cirrhosis and hepatocellular carcinoma (HCC) (1).

HBV is a small enveloped and partially double-stranded DNA virus belonging to the family *Hepadnaviridae* with a genome of approximately 3.2 kb in length that contains 4 overlapping open reading frames (ORFs): surface antigen (S), core protein (C), polymerase (Pol), and X protein (X). Due to its lack of proofreading ability, the HBV reverse transcriptase (RT) can induce HBV mutations at a higher frequency, resulting in the failure of antiviral therapy with nucleos(t)ide analogs (NAs) and liver disease progression due to persistent infection (2–6). The virus infects only hepatocytes, in which it builds a covalently closed circular (ccc) DNA template to generate progeny virions. The persistence of the viral genome owing to the cccDNA represents a major obstacle for developing a complete cure for a chronic HBV infection (7, 8). Most chronic hepatitis B patients require lifelong nucleos(t)ide analog (NA) treatment, which is highly effective in inhibiting viral replication and can reduce liver inflammation (9). However, due to persistent viral replication via cccDNA, NA treatment still cannot achieve a functional cure for a chronic infection, which is hallmarked by a sustained clearance of the circulating serum hepatitis B surface antigen (HBsAg) and an undetectable viral load in serum, and also leads to the emergence of drug-resistant strains of virus during long-term NA therapy (10, 11). Hence, there is an urgent need to develop new and more effective HBV immunotherapy to cure HBV chronic infections, ultimately leading to a sustained loss of HBsAg production. HBV immunotherapeutic vaccine approaches aim to break immune tolerance generated in HBV chronic infections by restoring effective cellular and humoral responses against HBV antigens, particularly impaired HBV specific T-cell responses (12, 13). To date, various HBV therapeutic vaccine modules based on HBsAg (14, 15), virus-like particles (VLPs) of HBsAg (16) and hepatitis B virus core antigen (HBcAg) (17), DNA (18) and peptides (19) have been used to eradicate HBV infections, but none of these have been in routine clinical use due to their various problems. One of the most important factors responsible for their failure is the lack of a sufficient immune boost to break immune tolerance at the T-cell level. Therefore, an emerging strategy for therapeutic vaccine development is to activate dendritic cells (DCs) via the use of proper immune adjuvant, resulting in recovery of the compromised immune responses in HBV carriers.

Type 1 interferon (IFN-I), with a strong anti-HBV activity, can also elicit DC maturation and augment cross presentation of antigens in DCs, resulting in NK cell-mediated and CTL-mediated antitumor or antiviral effects in mice (20). Recently, we reported that an HBV-derived 6-mer peptide, Poly6, exerts a strong anticancer effect in mice implanted with MC38 cells, a murine colon adenocarcinoma line, through the production of inducible nitric oxide synthase (iNOS)-producing DCs (Tip-DCs) in a type 1 interferon (IFN-I)-dependent manner (21), suggesting that Poly6, as an IFN-I inducer, may be a potential vaccine adjuvant for use in antivirus therapy, such as HBV therapeutic vaccines.

The aim of the present study was to evaluate the anti-HBV immunotherapeutic potential of a vaccine with Poly6 as an adjuvant in combination with HBsAg in an HBV transgenic mouse model. We confirmed that Poly6 can elicit DC maturation in both *in vivo* and *in vitro* systems in an IFN-I-dependent manner and that vaccination with Poly6 in combination with HBsAg can eliminate HBV virus and antigens in HBV transgenic mice via induction of both efficient HBV-specific cell-mediated and humoral immune responses.

## Materials and methods

### Animals

Six- to eight-week-old male HBV transgenic mice [IACUC number SNU-200918-5-2], type I IFN receptor α-chain knock-out (IFNAR KO) mice [IACUC number SNU-220401-3], and C57BL/6 mice were used throughout this study. HBV transgenic mice were generated by Macrogen, Inc., and C57BL/6 mice were purchased from Orient Bio (Seongnam, South Korea). IFNAR KO 129/SvEv mice (22) were kindly provided by Dr. Heung Kyu Lee (Korea Advanced Institute of Science and Technology) and backcrossed with C57BL/6J more than ten generations. All experiments utilizing animals in this study were approved by the Seoul National University Institutional Animal Care and Use Committee.

### Transgenic mouse model

Transgenic mice expressing HBV W4P mutant were generated by Macrogen, Inc and mice were interbred and maintained in pathogen-free condition at Macrogen, Inc (Seoul, Korea). Briefly, Pregnant Mare Serum Gonadotropin (PMSG, 7.5 IU) and human Chorionic Gonadotropin (hCG) were intraperitoneal injected every 48 hours with an interval (5 IU) to the C57BL/6N female mouse during 5-8 weeks for superovulation. After injection, these female mice were mated with C57BL/6N male mice. Next day, the female mice with virginal plug were sacrificed and harvested fertilized embryo and HBV W4P full genome was co-microinjected into one cell embryo using standard microinjection procedures for transgenic mice production (Macrogen, Seoul, Korea). After HBV DNA (4 ng/μl) for microinjection was directly injected into the male pronucleus of zygote by using micromanipulator, the microinjected embryos were incubated at 37°C during one or two hours. Fourteen to sixteen injected one cell staged embryo were transplanted by surgical methods into oviducts of the pseudopregnant recipient mice (ICR). After F0 were born, genotyping tested using tail cut samples for the presence of the transgene were performed and confirmed by PCR analysis of their genomic DNA PCR screening were done through phenol-extraction method. HBV TG mice were backcrossed with C57BL/6N more than ten generations. For genotyping, primers SF, 5’-TTG ACA AGA ATC CTC ACA ATA CC-3’ and SR, 5’-GGA GGT TGG GGA CTG CGA AT-3’, and HBsAg ELISA (Novus Biologicals, LLC, KA0286) were used. All experiments utilizing animals in this study were approved by the Seoul National University Institutional Animal Care and Use Committee (IACUC) and Macrogen Institutional Animal Care and Use Committee approval. The experimental animals were housed in the specific-pathogen-free Laboratory Animal Center.

### Immunization

The HBV TG mice, C57BL/6 mice, or IFNAR KO mice were randomly assigned to groups (n = 5). Mice were injected subcutaneously with 100 ul of PBS, HBsAg (SHB, 10 µg), HBsAg+Poly6 (10 µg each), or HBsAg (10 µg) + Alum (100 µg) mixtures following the immunization procedure on day 0 (Prime). For HBV TG mice, three subcutaneous boosts were administrated on day 7, 14, 28. For C57BL/6 mice and IFNAR KO mice, one subcutaneous boost was administrated on day 14.

### Safety assessment of Poly6

ICR mice were purchased from Orient Bio (Seongnam, South Korea). Four experimental groups were designed in this study: the PBS control group (sterile PBS) and the Poly6 experimental groups (5, 10, and 20 mg/kg). PBS or Poly6 was administered subcutaneously to 10 mice (5 males and females each) per group. Two weeks (Day 1, 2, 4, 8, and 15) of mortality, general symptom observation, weight change, and visual autopsy findings (adrenal gland, aorta, bone marrow, sternum, brain, coagulating gland, epididymis, esophagus, eye, gall bladder, harderian gland, heart, injection site, cecum. colon, duodenum, ileum, jejunum, rectum, kidney, liver, lung, lymph node, mesenteric, lymph node, mandibular, skeletal muscle, nerve optic, nerve peripheral, pancreas, pituitary gland, prostate gland, salivary gland, seminal vesicle, skin, spinal cord, thoracic, spleen, stomach) were observed and compared with the excipient control group. For statistical analysis, weight results were analyzed by ANOVA & Dunnett’s, normality was verified by Shapiro-Wilks test, and homogeneity of variance was tested by Leven test. In addition, a post-test was performed with Dunnett’s to confirm the group showing a significant difference from the excipient control group.

### HBV stock for infection

HepG2 cells were transfected with pHBV-1.2x vector containing the full-length HBV genotype C genome (2.5 μg) using lipofectamine 3000 (Thermofisher Scientific Inc., California, USA) and cultured for HBV replication. Supernatant was collected for every 3 days for 2 weeks and fresh medium was added. The supernatant was purified through a sterile 0.45-μm pore size filter and precipitated with 6% polyethylene glycol (PEG) 8000 overnight. The medium was ultracentrifuged, and the collected pellet was resuspended in PBS containing 15% fetal calf serum (FCS). After quantification by qPCR (SF, 5’-TTG ACA AGA ATC CTC ACA ATA CC-3’ and SR, 5’-GGA GGT TGG GGA CTG CGA AT-3’), 3 × 10^9^ HBV genome equivalents per milliliter were aliquoted and stored at −80°C.

### *In vitro* HBV neutralizing assay

An *in vitro* HBV neutralizing assay was performed using HepG2-NTCP-C4 cells. HepG2-NTCP-C4 cells were seeded in 96-well plates in DMEM/F-12 supplemented with GlutaMAX, 10% FBS, 100 U/ml PS, 10 mM HEPES, and 5 µg/ml insulin. The medium was changed to DMEM/F-12 with 10% FBS and 3% DMSO the next day, and the cells were cultured for an additional 20 h before infection. Mouse serum samples were incubated with the virus (3,000 GEq/cell) in the inoculation medium for one hour at 37°C before adding to cells. The inoculation was performed in DMEM/F-12 supplemented with 2% FBS, 4% PEG8000, and 3% DMSO. After a 20-hour inoculation, supernatant was removed, cells were washed three times with PBS, and 100 µl of fresh DMEM/F-12 supplemented with 2% FBS was added. The supernatant was harvested 6-7 days after infection for analysis of HBsAg secretion (HBsAg ELISA). Serum neutralization capacity was calculated as the relative percentage of infected HepG2-NTCP-C4 cells.

### Generation and stimulation of bone marrow-derived dendritic cells

Murine bone marrow cells were flushed out of the femurs and tibias of 7- to 10-week-old mice into complete medium, and a single cell suspension was made after passage through a cell strainer. Erythrocytes were lysed using red blood cell lysis buffer (Sigma-Aldrich, St. Louis, U.S.), and cells were washed with medium. BM cells were cultured in a 24-well plate with complete medium supplemented with 30 U/ml GM-CSF and 30 U/ml IL-4. Adherent cells were collected on Day 7, washed with medium, and used in assays.

### Flow cytometry

Single-cell suspensions were preincubated with Fc-receptor blocking solution (CD16/32) for 30 min and stained with FACS antibodies at 4°C for 1 h. All data were acquired on a BD FACS LSRFortessa X-20 and a BC FACSCalibur. The LSRII was analyzed with FlowJo software (BD Life Sciences, Franklin Lakes, NJ, USA).

### T cell proliferation assay

Six mice were injected subcutaneously with the HBsAg protein (10 μg/mouse). After 7 days, the splenocytes were isolated. The cells were subsequently stained with BV421-conjugated anti-CD3 (BD Biosciences), BV605-conjugated anti-CD4 (BD Biosciences), and PE-conjugated anti-CD8a (eBioscience, San Diego, CA, USA) for 30 min at 4°C and washed three times with ice cold FACS buffer. The BD FACS Diva 9.5.1 instrument (BD Biosciences) was used to sort the CD4 and CD8 T cell populations. One day before co-cultivation, BMDCs were also treated with the PBS, SHB, or SHB+Poly6 for 24 h. Proliferation assays were conducted using the fluorescent cytoplasmic tracking dye CFSE (Invitrogen, Carlsbad, CA, USA). The sorted CD4 and CD8 T cells were stained with 5 µM CFSE for 20 min at 37°C. And then, the CFSE-labeled T cells and BMDCs were co-cultured for 2 or 4 days. The cell cycle profiles were determined using LSRFortessa X-20 (BD Biosciences) and analyzed using Flowjo software.

### Isolation of liver immune cells

The liver was gently passed through a 200-gauge mesh and suspended in an RPMI medium. The cell suspension was centrifuged at 50 × *g* for 5 min, then, the supernatants were centrifuged at 500 × *g* for 5 min. Cell pellet was washed three times with PBS and purified using a Percoll gradient. The cells were resuspended in 40% Percoll in HBSS, and gently overlaid onto 70% Percoll and centrifuged at 800 g for 20 min. The cells were collected from the interface and washed twice with PBS.

### Footpad injection for DC migration tracking

For DC-migration tracking assay, BMDC were treated with PBS, Poly6, or LPS and were CFSE labeled. Then the CFSE-labeled DCs were injected into the left or right mouse footpad. 24 h later, the mice were sacrificed and the number of CFSE^+^ DCs migrated to the draining lymph nodes was analyzed by flow cytometry.

### Cells and reagents

HepG2-hNTCP-C4 cells were maintained in DMEM/F-12 supplemented with GlutaMAX, 10% FBS, 100 U/ml PS, 10 mM HEPES, 5 µg/ml insulin, and 500 µg/ml G418. The Poly6 (GRLVFQ) peptide from the HBV polymerase region overlapping with the preS1 region, was synthesized by the (9-fluorenylmethoxycarbonyl Fmoc)-based solid-phase method and characterized by Peptron Inc. (Cheongju, Korea) (**Supplementary Figure 1**).

### ELISA assay

The presence of anti-HBs antibody and anti-HBs titers were determined by ELISA (Alpha diagnostics Intl. Inc. Texas, USA and Abnoba, Taiwan) as per the manufacturer’s instructions.

### Immunofluorescence

Cells were seeded and cultivated in 6-chamber glass slides for 12 h before each experiment. The cells were fixed with 4% paraformaldehyde (PFA) and permeabilized with 0.25% Triton-X 100 for 10 min. After blocking with 5% goat serum, the cells were stained with anti-HBV core primary antibody (1:100, overnight at 4°C) and secondary antibodies (1:1000, 2 h at room temperature) in 1% bovine serum albumin (BSA) in PBST and mounted in mounting medium containing DAPI (VECTASHIELD, Vetor Laboratories, Inc., Burlingame, CA, USA). Images were captured and analyzed using software to quantify the staining intensity (Leica Software analysis, LAS X and ImageJ program, version 1.52a).

### Immunohistochemistry

The liver sections were fixed with 4% paraformaldehyde for 72 h in 4°C and embedded in paraffin. The embedded tissues were cut into 4∼6-μm-thick sections and deparaffinized with a xylene/ethanol solution. Antigen retrieval was performed with the heat-mediated method (sodium citrate buffer), and endogenous peroxidase was blocked by 3% H2O2. For IHC staining, the sections were incubated with primary and secondary antibodies, and chromogenic detection was developed through the conversion of 3,3′-diaminobenzidine (DAB) to a brownish precipitate that remained permanently detectable on slides and were visualized by light microscopy.

### H&E staining

Surgical specimens of the mouse liver tissues were paraffin-embedded for H&E staining. Paraffin-embedded tissue sections were deparaffinized and hydrated, then stained with hematoxylin and with eosin. The tissue sections were examined under a light microscope and scanned.

### Statistical analysis

The experimental data were analyzed with GraphPad Prism 9 (GraphPad Software, La Jolla, CA, USA). All experiments were independently repeated three times, and statistical analysis results are indicated in the figure legends. The *p value* indicating statistical significance was set at ^*^ *p* < 0.05, ^**^ *p* < 0.01, or ^***^ *p* < 0.001.

## Results

### Safety evaluation of the Poly6

Safety assessment was performed to determine the toxicity of Poly6 when subcutaneously injected into an ICR mouse (**Table 1**). Two weeks of mortality, general symptom observation, weight change, and visual autopsy findings were observed and compared with the control group (PBS). As a results, in all groups, no dead mice were observed, and the effect of the test substance on general symptoms, weight changes, and visual autopsy findings was not observed (**Table 2**; **Supplementary Table 1**). Based on the results, Poly6 did not elicit adverse or toxic effects in subcutaneously injected mice and the approximate lethal dose (ALD) under the test condition was estimated more than 20 mg/kg for both males and females.

**Table 1:** Safety assessment of Poly6 in subcutaneously injected ICR mice and composition of test groups and dosage.

Group	Sex	n	Mouse no.	Dose vol. (mL/kg)	Drug dose (mg/kg)
G1	M/F	5/5	1-5/21-25	10	0
G2	M/F	5/5	6-10/26-30	10	5
G3	M/F	5/5	11-15/31-35	10	10
G4	M/F	5/5	16-20/36-40	10	20

G1: Control (PBS), G2-G4: Poly6 administration groups.

**Table 2:** Safety assessment of Poly6 in subcutaneously injected ICR mice and body weights results.

			G1 0 mg/kg	G2 5 mg/kg	G3 10 mg/kg	G4 20 mg/kg
Body wt. (g)	1[a]	Mean SD N %Diff	32.85 0.63 5 -	33.71 1.52 5 2.26	33.53 1.43 5 2.1	33.65 0.91 5 2.4
	2[a]	Mean SD N %Diff	33.04 0.64 5 -	33.88 1.76 5 2.6	33.75 1.94 5 2.1	33.58 1.27 5 1.6
	4[a]	Mean SD N %Diff	33.71 0.97 5 -	34.58 1.93 5 2.6	34.20 1.64 5 1.5	33.89 1.18 5 0.6
	8[a]	Mean SD N %Diff	34.88 0.89 5 -	35.05 1.75 5 0.5	34.92 1.08 5 0.1	34.47 1.57 5 -1.2
	15[a]	Mean SD N %Diff	35.63 0.80 5 -	34.62 1.82 5 -2.8	35.12 0.87 5 -1.4	34.87 2.01 5 -2.1
Gain	1→ 15[a]	Mean SD N %Diff	2.78 0.46 5 .	0.91 0.71 5 -67.3	1.58 1.29 5 -43.0	1.22 1.43 5 -55.9

[a] - Anova & Dunnett.

### Poly6 promoted the maturation and migration capacity of DCs

**Figure 1:**
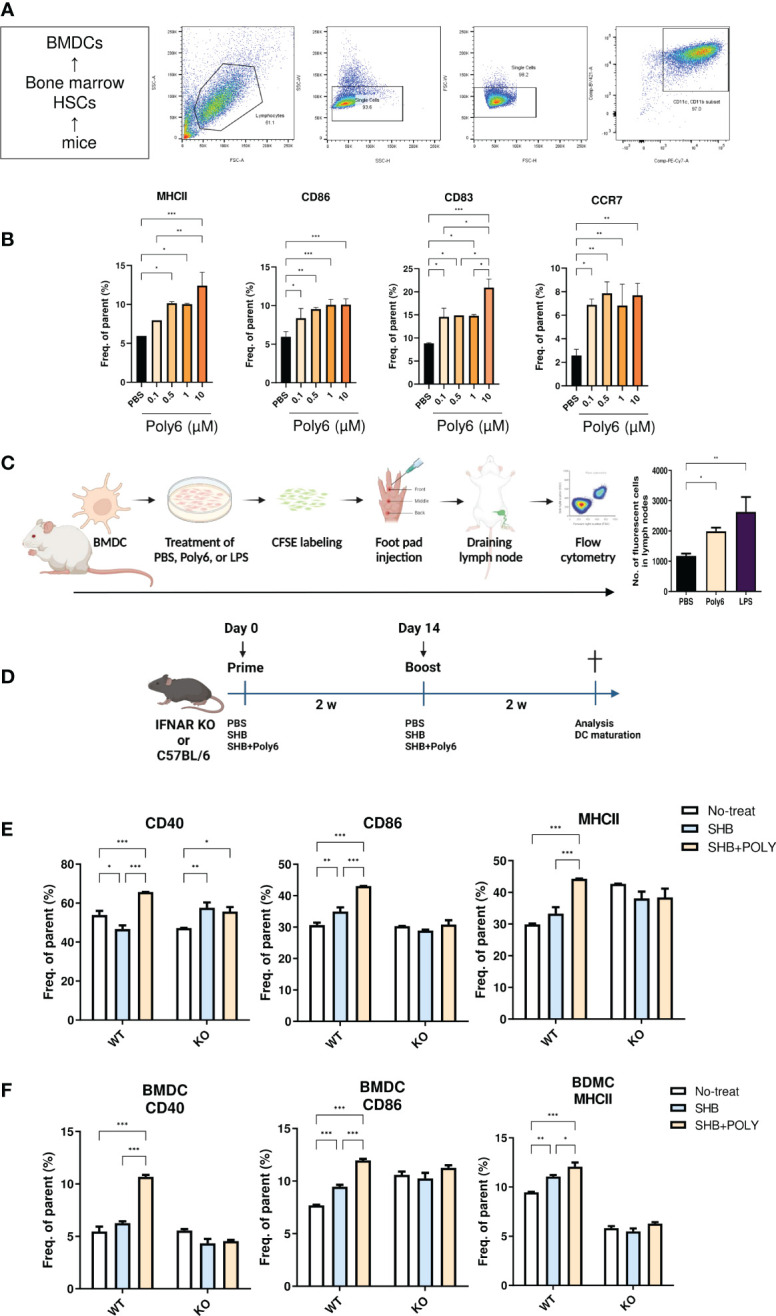
Poly6 promoted the maturation and migration capacity of DCs. **(A)** The gating strategy of DCs isolated from BMDCs. **(B)** Surface expression of costimulatory molecules and maturation markers on BMDCs upon exposure to Poly6. **(C)** Schematic illustration of footpad DC injection and the CFSE-based migration assay. The draining lymph node was isolated, and CFSE-labeled cells were characterized by flow cytometry. **(D)** Schematic illustration of the vaccination protocol in IFNAR KO and C5BL/6 mice. **(E)** Flow cytometry results showing the expression of CD40, CD86, and MHCII on splenic mDCs in IFNAR KO mice and C57BL/6 mice. **(F)** Flow cytometry results showing the expression of CD40, CD86, and MHCII on BMDCs in IFNAR KO mice and C57BL/6 mice. The experiments were repeated three times to demonstrate replicability. The results were evaluated for statistical significance by one-way ANOVA with Tukey’s *post hoc* test. Differences were considered significant when *p < 0.05, **p < 0.01, and ***p < 0.001.

As professional APCs, DCs play a crucial role in facilitating antigen-specific T-cell responses in chronic HBV infection (23, 24). As our previous study revealed that Poly6 exerted a strong anticancer effect in tumor-implanted mice via iNOS-producing DCs (21), we examined whether Poly6 itself could promote the maturation and migration capacity of DCs in BMDCs (**Figure 1**; **Supplementary Figure 2**). The murine DC population was selected in high purity (**Figure 1A**), and MHCII, CD86, CD83, and CCR7 were evaluated by flow cytometry 24 h poststimulation. As shown in **Figure 1B**, Poly6 treatment upregulated the costimulatory molecules CD86 and MHC class II as well as CD83 which is known to a pivotal factor during the differentiation of T and B cells (25) in BMDCs in a dose-dependent manner. In addition, CCR7, which helps DCs migrate into T-cell zones in lymph nodes to initiate antigen presentation and T-cell activation (26), was upregulated by Poly6 treatment (**Figure 1B**; **Supplementary Figures 2, 3**). Therefore, to further investigate DC migration capacity by Poly6, we performed a migration assay using mouse footpad injection. BMDCs treated with PBS, Poly6, or LPS were labeled with CFSE and injected into mouse footpads. CSFE-labeled DCs that had drained to lymph nodes were analyzed by flow cytometry. As shown in **Figure 1C**, DCs treated with Poly6 showed increased migration capacity compared to the control group. Taken together, our results indicated that Poly6 efficiently promoted the maturation and migration ability of DCs, suggesting its potential as a vaccine adjuvant.

### Vaccination with Poly6 in combination with HBsAg induced DC maturation in a type I IFN-dependent manner

Type I interferons (IFNs) play a role in bridging the innate and adaptive immune responses. In particular, IFNs promote DC activation and subsequent CD4+ and CD8+ T-cell priming (27). As we had verified that Poly6 induced strong immune responses through type I IFN signaling in our previous studies (21, 28), in this study, we examined whether vaccination with Poly6 in combination with HBsAg facilitated DC maturation in a type I IFN-dependent manner by comparing DC activities between C57BL/6 and IFNAR KO mice. Six- to eight-week-old C57BL/6 or IFNAR KO mice were vaccinated in a prime-boost regimen (**Figure 1D**), and mouse splenocytes were assessed for DC maturation by flow cytometry. As shown in **Figure 1E**, in C57BL/6 mice, the costimulatory molecules CD40 and CD86 and the MHC class II surface activating marker were significantly upregulated in the group vaccinated with Poly6 in combination with HBsAg compared to the control groups. Meanwhile, vaccination with Poly6 in combination with HBsAg failed to activate DC maturation in IFNAR KO mice. Consistently, vaccination with Poly6 in combination with HBsAg significantly elevated surface expression levels of CD40, CD86, and MHC class II in WT BMDC, whereas maturation of IFNAR KO BMDC was not affected by Poly6 (**Figure 1F**; **Supplementary Figure 4**). These results indicated that Poly6 contributes to DC maturation in a type I IFN-dependent manner in mice subjected to vaccination with Poly6 in combination with HBsAg.

### Poly6 addition into the alum-adjuvanted HBsAg vaccine protocol potentiated HBsAg-specific cellular and humoral immune responses in C57BL/6 mice

**Figure 2:**
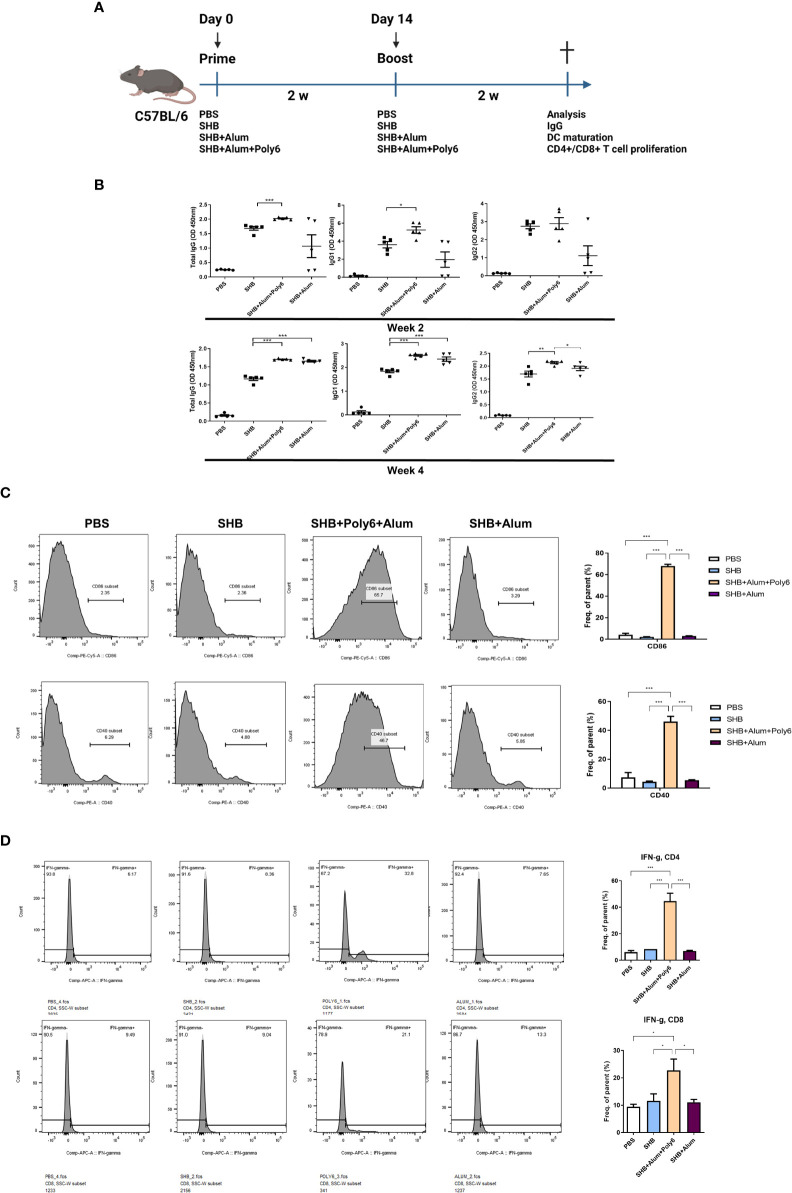
Poly6 addition to the alum-adjuvanted HBsAg vaccine protocol potentiated HBsAg-specific cellular immune responses in C57BL/6 mice. **(A)** Schematic illustration of the immunization regimen. C5BL/6 mice were immunized with PBS, SHB (10 µg), SHB (10 µg) +Alum (100 µg), or SHB (10 µg) + Alum (100 µg) + Poly6 (10 µg) as shown (n=5 in each group). **(B)** Serum anti-HBsAg total IgG, IgG1, and IgG2 were analyzed at weeks 2 and 4 after the first immunization. **(C)** Flow cytometry results showing the expression of CD86 and CD40 on splenic mDCs. **(D)** The proportion and abundance of IFNγ-producing CD4^+^ or CD8^+^ cells. The experiments were repeated three times to demonstrate replicability. The results were evaluated for statistical significance by one-way ANOVA with Tukey’s *post hoc* test. Differences were considered significant when *p < 0.05, **p < 0.01, and ***p < 0.001.

The lack of a cell-mediated or T helper 1 (TH1) immune response to alum adjuvants necessitates the use of efficient adjuvants for HBV-specific vaccine formulations (29). Therefore, we sought to investigate whether the addition of Poly6 to alum-adjuvanted HBsAg resulted in strong cellular and humoral immune responses in C57BL/6 mice. Seven-week-old mice were vaccinated in a prime-boost regimen (**Figure 2A**), and serum samples collected from the mice were evaluated for HBsAg-specific IgG and IgG subtype by ELISA. As a result, the mean titers of HBsAg-specific total IgG, IgG1, and IgG2 were significantly increased in the SHB+alum+Poly6 combination group at 2 weeks (**Figure 2B**). At 4 weeks, the SHB+alum combination group showed increased IgG levels similar to the SHB+alum+Poly6 group, indicating that the combination of Poly6 enhanced HBsAg-specific humoral immunity in mice. Next, we examined whether Poly6 addition to alum-adjuvanted HBsAg induced the maturation of DCs in the spleens of mice. Our data indicated that the spleens of SHB+alum+Poly6 group mice had a dramatically activated expression of the costimulatory molecules CD86 and CD40 (**Figure 2C**). We then assessed whether Poly6 addition to alum-adjuvanted HBsAg enhanced the induction of HBsAg-specific cell-mediated immune responses. Vaccination of mice with HBsAg+alum+Poly6 significantly increased the expression of IFN-γ by both CD4^+^ (Th1) and CD8^+^ T cells (Tc1) (**Figure 2D**). Notably, the group with alum in combination with HBsAg failed to stimulate HBsAg-specific cell-mediated immune responses, indicating a crucial role of Poly6 addition in the induction of the HBsAg-specific cell-mediated immune response. Taken together, these results suggested that Poly6 could contribute to inducing efficient HBV-specific humoral and cellular immune responses as an adjuvant for HBsAg-based vaccines, possibly via DC activation, in a C57BL/6 mouse model (**Figure 2**; **Supplementary Figure 5**).

### Vaccination with Poly6 in combination with HBsAg induced DC maturation and robust cell-mediated responses in HBV transgenic mice

**Figure 3:**
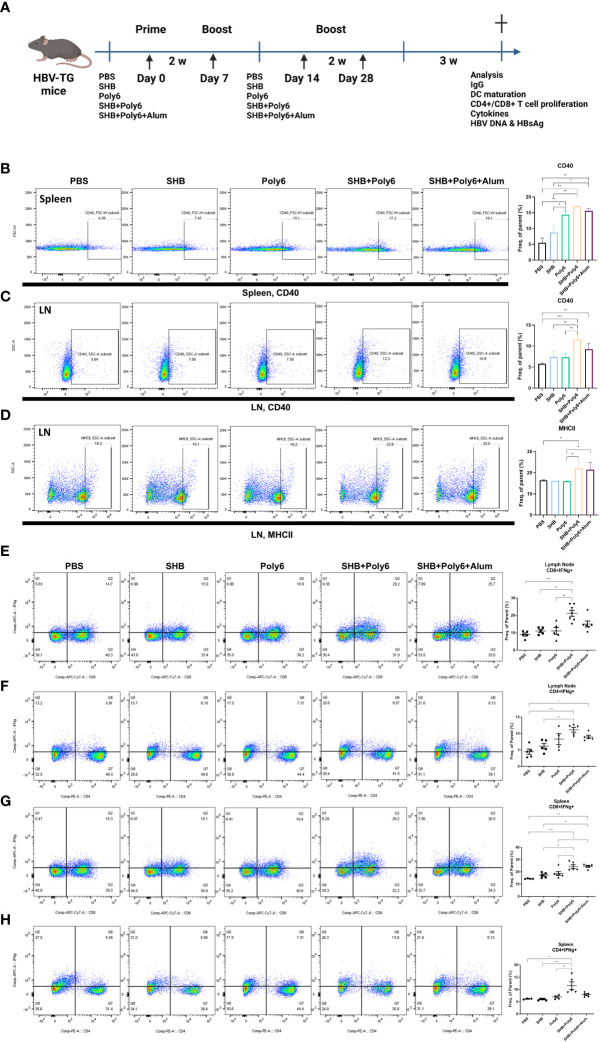
Vaccinations containing Poly6 promoted DC maturation and intensified potent Ag-specific CD4+ and CD8+ T-cell responses in transgenic mice. **(A)** Schematic illustration of the vaccination protocol in HBV-transgenic mice (n=5 in each group). **(B-D)** Surface expression of DC maturation markers by splenic and lymphatic DCs. **(E-F)** The proportion and abundance of IFNγ-producing CD4^+^ or CD8^+^ cells in the lymph nodes. **(G-H)** The proportion and abundance of IFNγ-producing CD4^+^ or CD8^+^ cells in the spleens. The experiments were repeated three times to demonstrate replicability. The results were evaluated for statistical significance by one-way ANOVA with Tukey’s *post hoc* test. Differences were considered significant when *p < 0.05, **p < 0.01, and ***p < 0.001.

**Figure 4:**
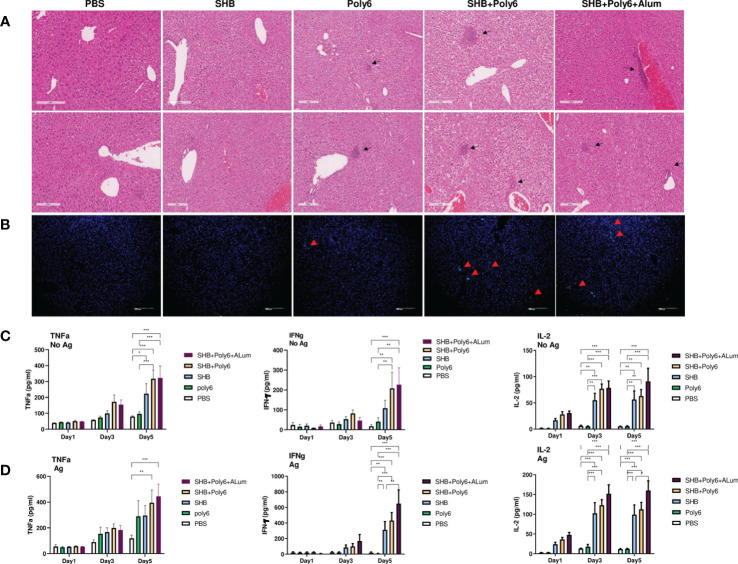
Vaccinations containing Poly6 induced potent stimulation of proinflammatory cytokines. **(A, B)** Histopathology of HBV transgenic mouse livers. Liver tissues were obtained, fixed, sectioned, and stained with H&E **(A)** and for CD8+ T cells **(B)**. Bar = 200 µm. **(C, D)** Cytokine release in splenocyte culture supernatant upon *in vitro* stimulation with or without HBV SHB antigen. TNFα, IFNγ, and IL-2 were assessed by ELISA. Concentration levels are expressed as the mean ± SEM of five mice for each group of mice. The results were evaluated for statistical significance by one-way ANOVA with Tukey’s *post hoc* test. Differences were considered significant when *p < 0.05, **p < 0.01, and ***p < 0.001.

**Figure 5:**
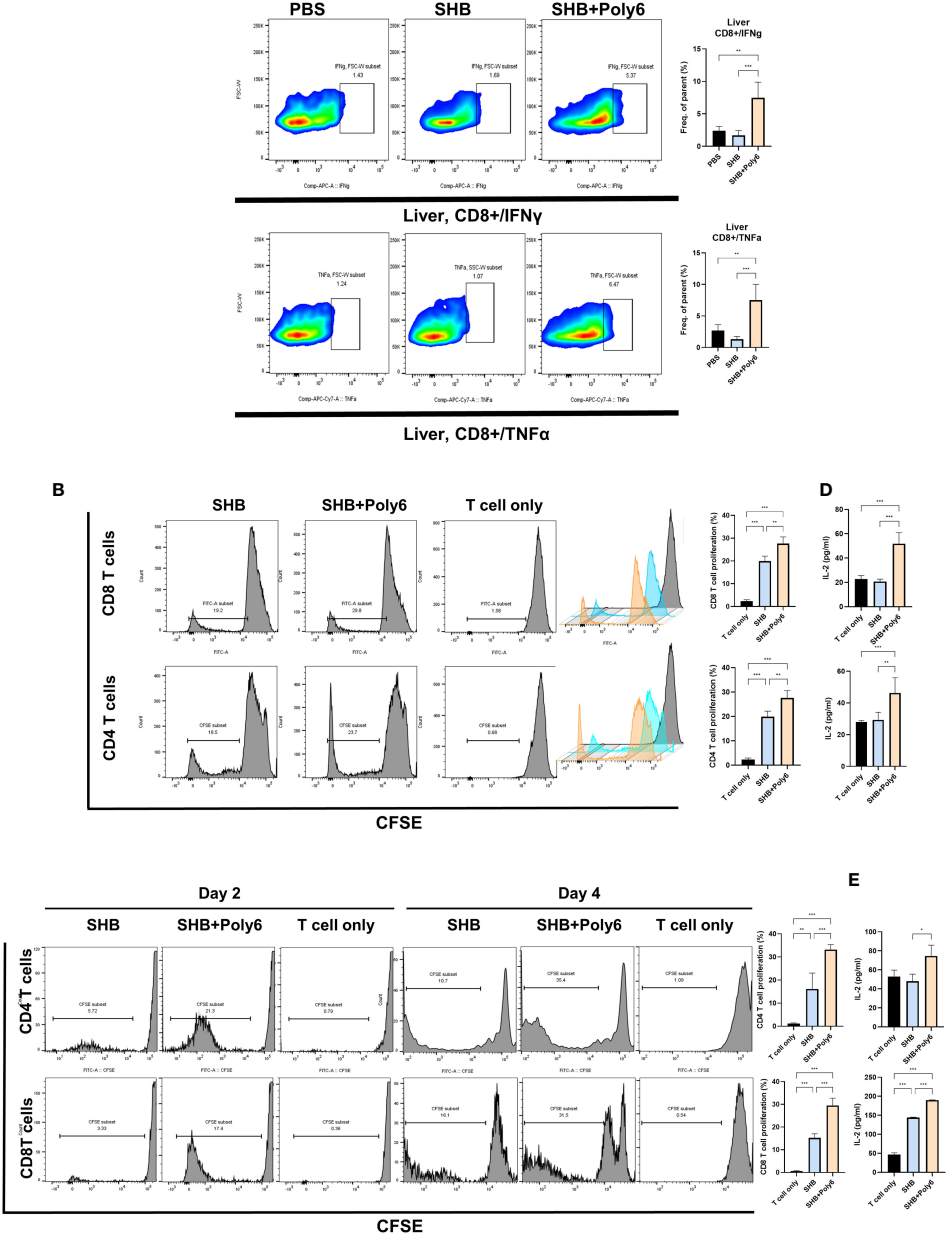
Vaccinations containing Poly6 elicited enhanced T cell proliferation and induced robust cell-mediated responses in HBV transgenic mice liver. **(A)** The proportion and abundance of IFNγ- and TNFα-producing CD8^+^ cells in the liver tissues. **(B)** Flow cytometric analysis of the proliferation of CFSE-labeled CD4 and CD8 T cells, which were isolated from WT mice, following the treatment of BMDCs with Poly6-combined HBsAg. **(C)** Flow cytometric analysis of the proliferation of CFSE-labeled CD4 and CD8 T cells, which were isolated from HBV TV mice. **(D)** Determination of IL-2 levels released in the supernatants of CD4 and CD8 T cells, which were isolated from WT mice, co-cultured with BMDCs. **(E)** Determination of IL-2 levels released in the supernatants of CD4 and CD8 T cells, which were isolated from HBV TG mice, co-cultured with BMDCs. The results were evaluated for statistical significance by one-way ANOVA with Tukey’s *post hoc* test. Differences were considered significant when *p < 0.05, **p < 0.01, and ***p < 0.001.

Given that vaccination with Poly6 in combination with HBsAg resulted in both higher humoral immune responses and stronger cell-mediated immune responses in C57BL/6 mice, we sought to determine whether the combined Poly6/HBsAg vaccination protocol could stimulate HBV-specific cell-mediated immune responses in HBV transgenic mice (**Figure 3A**). First, we examined the maturation of DCs. As shown in **Figures 3B–D** and **Supplementary Figure 6**, the expression of CD40 and MHCII was upregulated on DCs in the spleens and LNs of the Poly6/HBsAg combination groups irrespective of alum addition. We then assessed whether Poly6 combined with HBsAg could amplify the induction of CD4^+^ and CD8^+^ T-cell responses in HBV transgenic mice. As shown in **Figures 3E–H**, transgenic mice subjected to vaccination with Poly6 in combination with HBsAg exhibited increased IFN-γ-expressing CD4^+^ (Th1) and CD8^+^ T (Tc1) cells in their LNs and spleens. In addition, there was a strong association with lymphocyte infiltration found in the livers of the mice vaccinated with the Poly6/HBsAg combination compared to the PBS or SHB groups (**Figure 4A**; **Supplementary Figure 7**). Consistently, the infiltration of CD8^+^ T cells in the livers was also observed in the mouse groups vaccinated with the Poly6/HBsAg combination but not in the other groups (**Figure 4B**). Then, we measured Th1 cytokines (IL-2, IFN-γ and TNFα) in the culture supernatants of vaccinated HBV transgenic mouse splenocytes (**Figure 4**; **Supplementary Figure 7**). We observed significantly increased TNFα, IFN-γ, and IL-2 secretion levels in splenocytes in the groups vaccinated with the Poly6/HBsAg combination versus the other groups (**Figure 4C**), and HBs and HBc protein stimulation intensified the magnitude of the responses (**Figure 4D**), indicating that Poly6 potentiated Ag-specific Th1 responses. As it is widely recognized that central immune tolerance to HBV occurs in HBV TG mice liver (30), it is necessary to break immune tolerance in the liver. We sought to determine whether the combined Poly6/HBsAg vaccination protocol could induce HBV-specific CD8+ and CD4+ T cells secreting cytokines such as IFN-γ and TNFα in TG mice liver, which is weakly expressed in CHB patients and HBV TG mice (31). As shown in **Figure 5A** and **Supplementary Figure 8**, vaccination with Poly6 in combination with HBsAg exhibited increased IFN-γ- and TNFα-expressing CD4^+^ (Th1) and CD8^+^ T (Tc1) cells in their liver tissues. Taken together, these results indicated that vaccination with Poly6 in combination with HBsAg induced DC maturation and potentiated HBV-specific cellular immune responses in HBV transgenic mice.

### BMDCs treated with Poly6 in combination with HBsAg elicited enhanced T cell proliferation in mice immunized with HBsAg

To evaluate the stimulation ability of Poly6-treated DC, we performed T cell proliferation assay, in which CFSE-labeled T cells were used. After 2 or 4 days incubation, CFSE-labeled T cells were collected for flow cytometry. As shown in **Figures 5B, C**, BMDCs treated with Poly6 in combination with HBsAg induced significantly higher levels of CD4 and CD8 T cell proliferation than the BMDCs that were treated with HBsAg or PBS, in T cells isolated from both WT (**Figure 5B**) and HBV TG (**Figure 5C**) mice. Consistently, IL-2 secretion levels in stimulated CD4 and CD8 T cells were significantly increased in Poly6/HBsAg combination group (**Figures 5D, E**) indicating enhanced T cell proliferation capacity in Poly6/HBsAg vaccination.

### Vaccination with Poly6 in combination with HBsAg induced effector memory T cells

**Figure 6:**
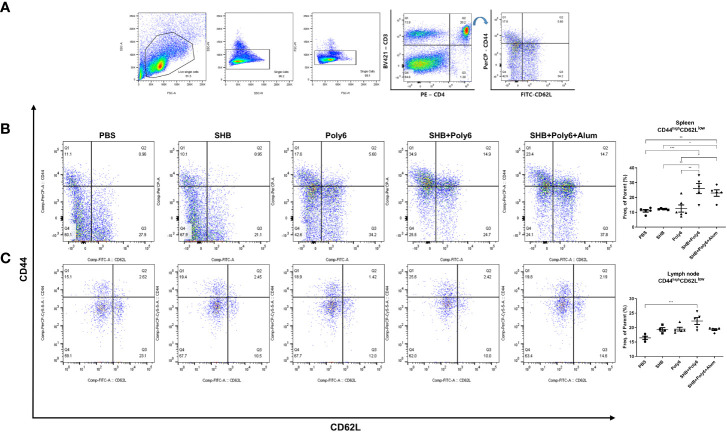
Vaccinations containing Poly6 induced effector memory T cells. **(A)** The gating strategy of effector memory T cells (T_EM_) in spleen and lymph node. **(B, C)** The proportion and abundance of CD44^high^CD62L^low^ CD4 T cells in spleen and lymph node. The results were evaluated for statistical significance by one-way ANOVA with Tukey’s *post hoc* test. Differences were considered significant when *p < 0.05, **p < 0.01, and ***p < 0.001.

T-cell memory is the fundamental goal of vaccination for faster and stronger secondary responses to subsequent antigen encounters (32); in particular, effector memory T cells (T_EM,_ CD44^high^/CD62L^low^) are considered to be efficient at producing a secondary generation of memory T cells. Therefore, we investigated whether Poly6-combined HBsAg vaccination produced an effector memory T-cell population for long-term memory by flow cytometry (**Figure 6A**). As shown in **Figures 6B, C**, we observed a remarkable induction of CD44^high^/CD62L^low^ CD4^+^ cells in spleen and lymph nodes of groups vaccinated with Poly6 in combination with HBsAg compared to other groups. Overall, the production of antigen-specific effector memory CD4+ T cells after vaccination, which is crucial to achieve an appropriate humoral response, was significantly increased in the group vaccinated with Poly6 in combination with HBsAg, suggesting the feasibility of Poly6 as an adjuvant for an HBV therapeutic vaccine.

### Vaccination with Poly6 in combination with HBsAg led to enhanced production of virus-neutralizing antibodies and exerted a strong anti-HBV effect in transgenic mice

**Figure 7:**
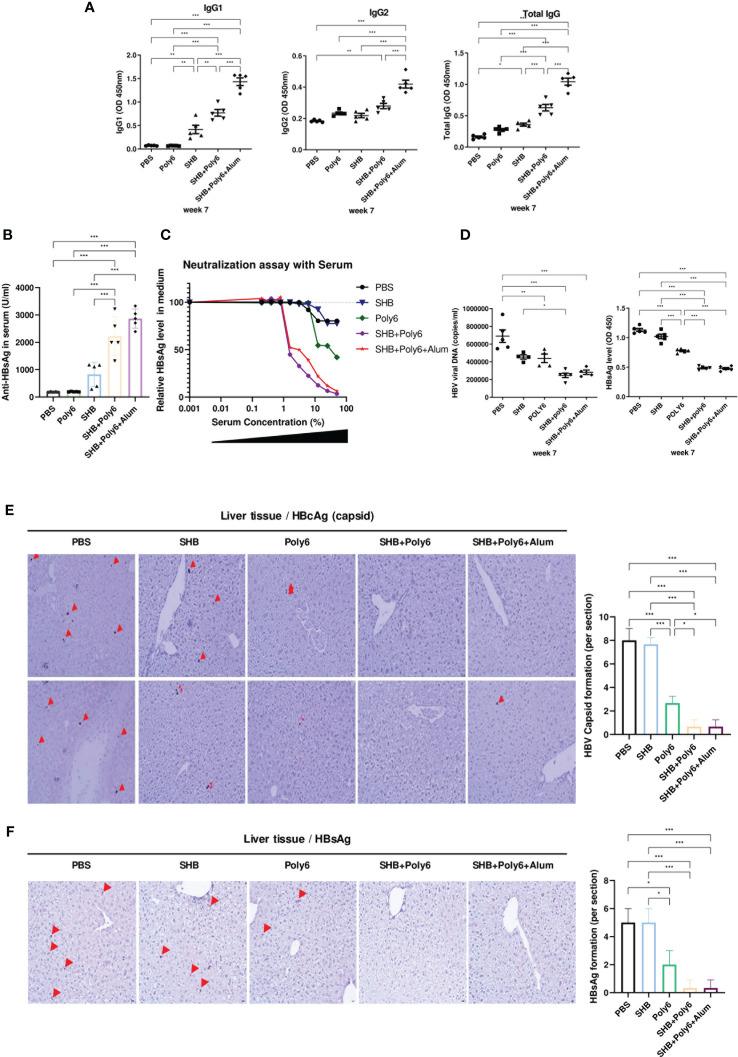
Immunization with Poly6 in combination with HBsAg produced virus-neutralizing antibodies and efficiently eliminated HBV in transgenic mice. **(A)** Serum anti-HBsAg total IgG, IgG1, and IgG2 were analyzed at week 7 after the first immunization. **(B)** Anti-HBsAg Abs in serum (U/ml) were measured by ELISA. **(C)** HBV neutralization by transgenic mouse serum. The results are shown as percent of infection in the presence of the indicated concentrations of serum samples as measured by an ELISA for HBsAg in medium in HepG2-NTCP-C4 cells. **(D)** HBsAg and HBV viral DNA in transgenic mouse serum were measured by ELISA and qPCR, respectively, at week 7. **(E)** Immunohistochemical (IHC) analysis of HBcAg (arrow) *in vivo*. Liver tissue from the transgenic mice was stained for HBcAg, and representative fields are shown. **(F)** Immunohistochemical (IHC) analysis of HBsAg (arrow) *in vivo*. Liver tissue from the transgenic mice was stained for HBsAg, and representative fields are shown. The results were evaluated for statistical significance by one-way ANOVA with Tukey’s *post hoc* test. Differences were considered significant when *p < 0.05, **p < 0.01, and ***p < 0.001.

We next evaluated HBsAg-specific humoral immune responses in immunized HBV-transgenic mice. As shown in **Figure 7A** and **Supplementary Figure 9**, IgG levels against HBsAg were significantly increased in the group vaccinated with Poly6 in combination with HBsAg and were even higher than those in the group vaccinated with Poly6 and alum in combination with HBsAg, as alum is known to potentiate humoral immunity (33). We then measured the amount of anti-HBsAg Abs in serum, and the groups vaccinated with HBsAg in combination with Poly6 or Poly6-alum contained more than 2000 U/ml anti-HBsAg Abs (**Figure 7B**). To examine the neutralizing capacity of the produced anti-HBsAg Abs in the vaccinated groups, we performed an *in vitro* HBV neutralizing assay using HepG2-NTCP-C4 cells (**Figure 7C**). As expected, serum antibodies from groups vaccinated with HBsAg in combination with Poly6 or Poly6+alum efficiently neutralized HBV infection in the HepG2-NTCP-C4 infection assay, indicating that Poly6 induced a robust humoral immune response. Given that vaccination with Poly6 in combination with HBsAg resulted in both strong cell-mediated immune responses and efficient humoral immune responses in HBV-transgenic mice, its potential for therapeutic vaccination was examined. As shown in **Figure 7D**, serum HBV DNA and HBsAg levels in the groups vaccinated with HBsAg in combination with Poly6 or Poly6+alum were significantly reduced at 7 weeks after the first immunization. In addition, intrahepatic HBcAg and HBsAg were also reduced in the two groups vaccinated with Poly6 in combination with HBsAg compared to the other groups (**Figures 7E, F**; **Supplementary Figures 10, 11**). Taken together, these results suggested that Poly6 could not only stimulate the production of anti-HBsAg Abs with neutralizing activity but also exert an anti-HBV therapeutic effect as an adjuvant for HBsAg vaccination irrespective of alum addition.

## Discussion

Although current direct-acting NA agents are effective in inhibiting viral replication and limiting HBV-associated disease progression, they rarely result in a functional cure for chronic HBV infections (34), and cessation of treatment can result in a rebound of viremia (35). Immune intervention using interferon-α is effective only in a small subset of HBV carriers and is not particularly effective in CHB patients with genotype C, which is endemic in Asian nations, including China, Japan and South Korea (36). For a functional cure of chronic HBV infections, induction of HBV-specific T-cell activation is necessary, which can be achieved through the use of immunotherapeutic agents capable of breaking the systemic immune tolerance in chronic carriers (37, 38). Dendritic cells (DCs), major antigen-presenting cells, can cross-present extracellular antigens to CD8+ T cells and induce cytotoxic T lymphocyte (CTL) responses (39). Therefore, the use of new adjuvants capable of activating DC maturation and cross presentation is required for the development of an HBV therapeutic vaccine to eradicate HBV infection.

Previously, we reported that a 6-mer peptide (Poly6) of the overlapping polymerase corresponding to a preS1 deletion associated with liver disease progression in Korean chronic patients infected with genotype C2 (40–42) can exert antiviral effects against HIV-1 and SARS-CoV-2 by inhibiting the viral integrase via epigenetic modification (43) and by eliciting IFN-I production (28), respectively, suggesting the role of Poly6 as an inducer of IFN-I capable of eliciting antiviral effects. Since IFN-I can induce DC activation and cross presentation (20), Poly6 is also expected to play a crucial role as an adjuvant in antiviral and anticancer therapeutic vaccine modules. We recently reported that Poly6 could elicit a strong anticancer immune response via the production of TNF/iNOS-producing dendritic cells (Tip-DCs) in an IFN-I-dependent manner in a tumor-bearing mouse model (21). Therefore, in this study, we sought to evaluate the potential of Poly6 as an HBV therapeutic vaccine adjuvant in an HBV-transgenic mouse model.

First, we investigated whether Poly6 could exert synergistic adjuvant potential with alum in vaccinated C57BL/6 mice by assessing the HBsAg-specific humoral responses and CMI. Our data indicated that Poly6 addition to an alum-adjuvanted HBsAg vaccination can strengthen HBsAg-specific CMI responses (**Figure 2**), which is the major weakness of alum-based protein vaccination (44), via increased DC maturation or migration capacity in an IFN-I-dependent manner (**Figures 1E, F**), suggesting that Poly6 addition to the conventional alum-adjuvanted HBsAg vaccine protocol can not only further strengthen the effect of conventional preventive HBV vaccines but also act as a new therapeutic vaccine for HBV treatment.

Second, we evaluated the potential of Poly6 as an adjuvant of a therapeutic vaccine in a transgenic mouse model. Our data indicated that vaccination with Poly6-adjuvanted HBsAg, irrespective of alum addition, can exert a strong anti-HBV effect by eliciting the production of HBV-specific CMI and humoral immune responses with strong anti-HBV neutralizing activity (**Figure 7**). Although alum-adjuvanted HBsAg vaccination can also lead to HBsAg-specific IgG production almost comparable to that of the vaccine module containing Poly6, its neutralizing potential cannot achieve that of the vaccines containing Poly6 (**Figure 7C**), suggesting that Poly6 addition could compensate for the lack of T helper cells in Ab production of the former. Moreover, enhanced production of the HBV-specific effector memory T-cell population (**Figure 6**) further supports the feasibility of Poly6 as an adjuvant for an HBV therapeutic vaccine. Together, these results suggest that Poly6 could contribute to strengthening the therapeutic potential of HBV mainly by restoring impaired HBV-specific T-cell-mediated immune responses generated during chronic infections, which might be primarily mediated by activating DC maturation.

In conclusion, our data indicated that an HBV-derived peptide, Poly6, leads to successful suppression of HBV virus and antigens to a low level in HBV transgenic mice as an adjuvant of the HBV therapeutic vaccine, which is primarily mediated by activation of DC maturation in an IFN-I-dependent manner, resulting in elicitation of HBV-specific CMI and humoral immune responses. Furthermore, vaccination with Poly6 in combination with HBsAg can also lead to increased effector memory T cells (T_EM_). Therefore, a vaccine with Poly6 in combination with HBsAg is an efficient and promising candidate for an HBV therapeutic vaccine.

## Data availability statement

The original contributions presented in the study are included in the article/supplementary material. Further inquiries can be directed to the corresponding author.

## Ethics statement

The animal study was reviewed and approved by Seoul National University Institutional Animal Care and Use Committee.

## Author contributions

Y-MC and B-JK contributed to the conception and design of the study and performed the statistical analysis. Y-MC, DK, and JJ organized the database. Y-MC, DK, and JJ performed the lab work. Y-MC and B-JK wrote and revised the manuscript. All the authors read and approved the submitted version.
