# Supplementary material for: A hepatitis B virus-derived peptide combined with HBsAg exerts an anti-HBV effect in an HBV transgenic mouse model as a therapeutic vaccine
Source: Front Immunol. 2023 Jun 2;14:1155637. doi: 10.3389/fimmu.2023.1155637 (PMC10272379; doi:10.3389/fimmu.2023.1155637)
Supplement: Supplementary file 1 [file DataSheet_1.docx]

Supplementary Material

A hepatitis B virus-derived peptide combined HBsAg induces exerts an anti-HBV effect in HBV transgenic mouse model as a therapeutic vaccine First Author*, Co-

Yu-Min Choi^1†^, Dong Hyun Kim^1†^, Junghwa Jang^1^, and Bum-Joon Kim^1,2,3,4,5^

*** Correspondence:** Bum-Joon Kim: [kbumjoon@snu.ac.kr](mailto:kbumjoon@snu.ac.kr)

## Supplementary Figures

| (A) | 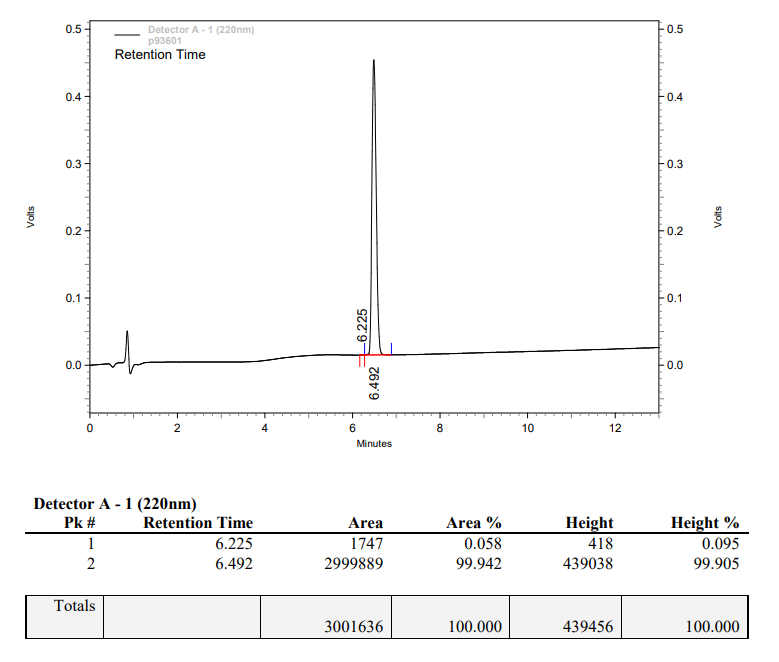 |
| --- | --- |
| (B) | 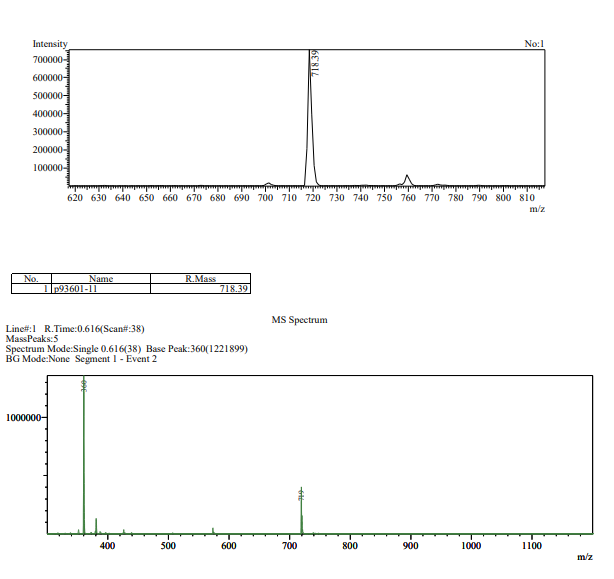 |

**Supplementary Figure 1 (A) Area % report (B) MS Data report** The Poly6 (GRLVFQ) peptide was synthesized by the Fmoc (9-fluorenylmethoxycarbonyl)-based solidphase method. The area percent report and MS data report was performed

| **CD83** | **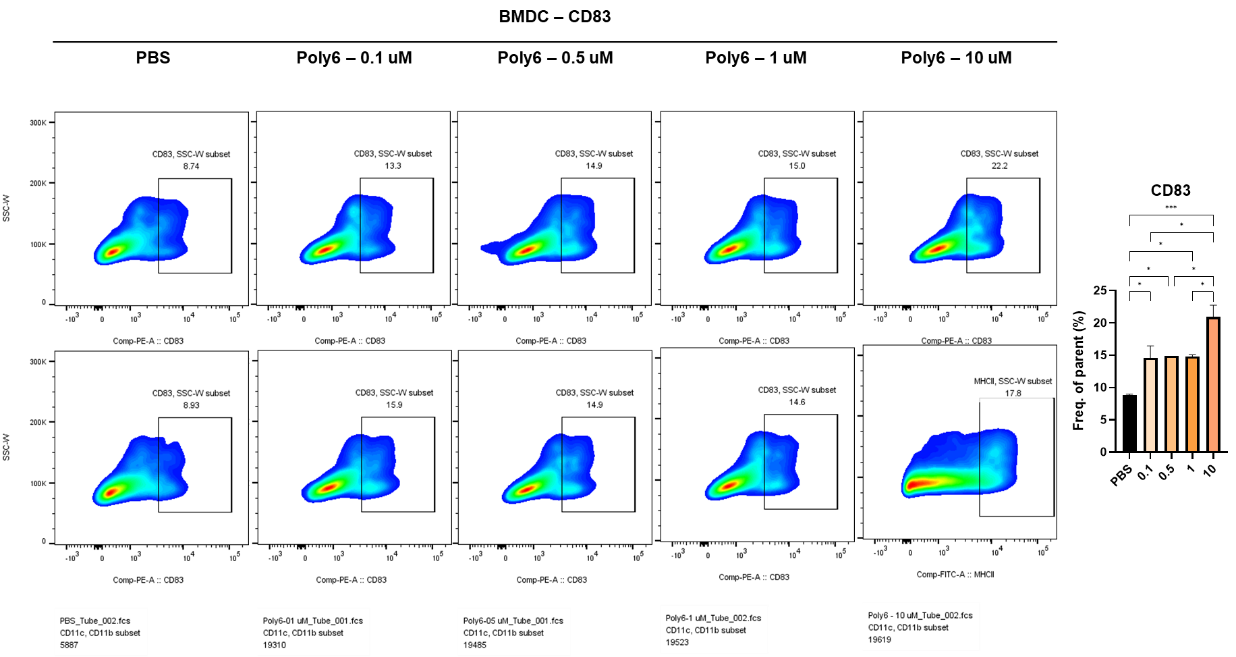** |
| --- | --- |
| **CD86** | **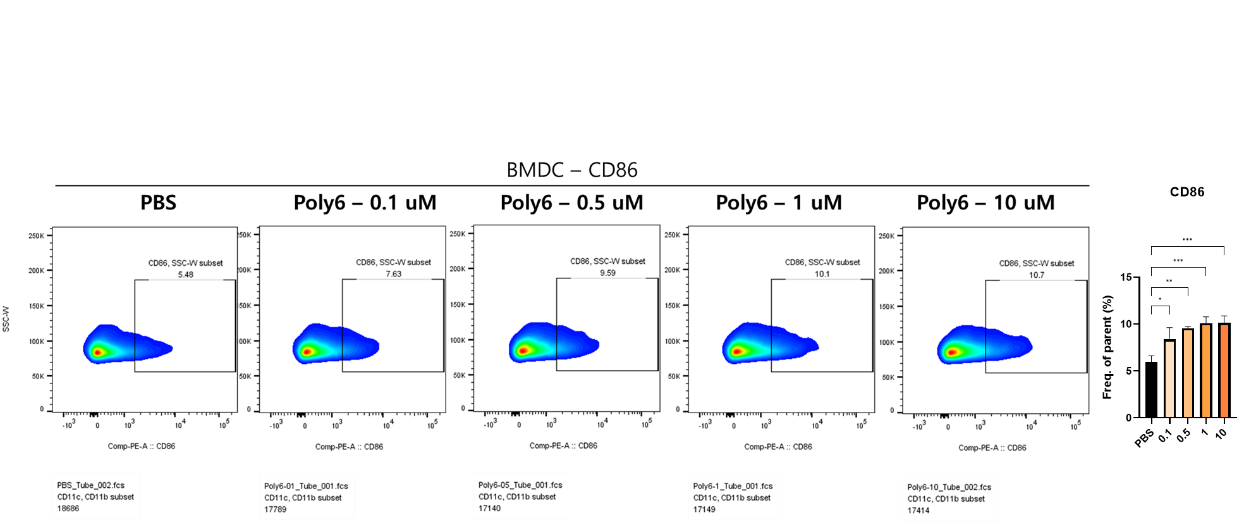** |
| **MHCII** | **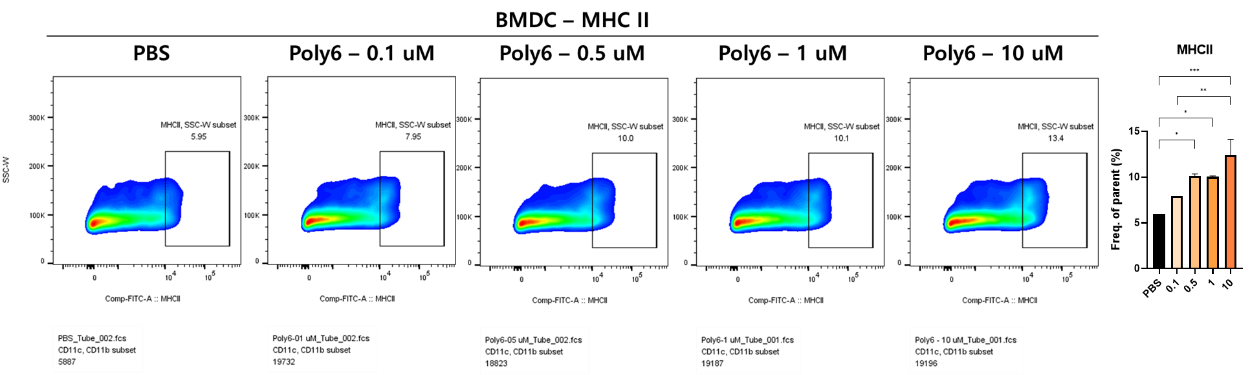** |
| **CCR7** | **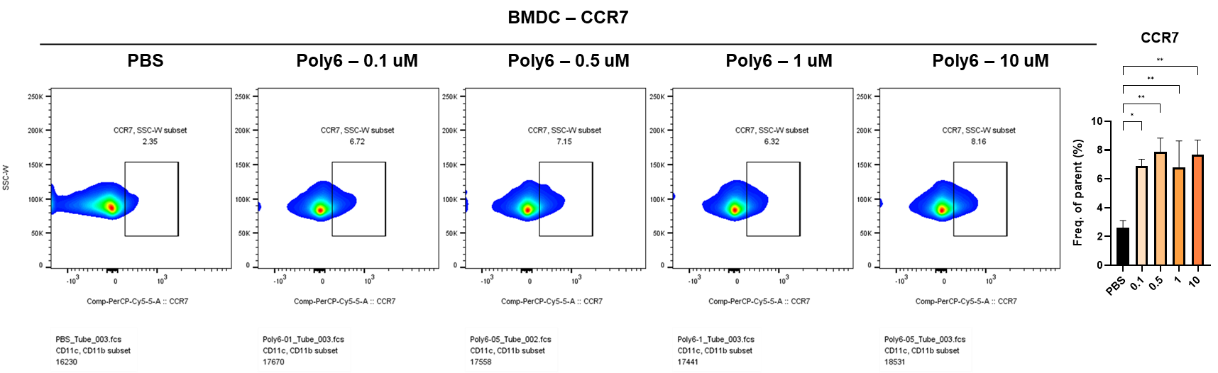** |

**Supplementary Figure 2. Poly6 promoted the maturation and migration** **capacity of DCs** Surface expression of co-stimulatory molecules and maturation markers by BMDCs upon exposure to Poly6

| **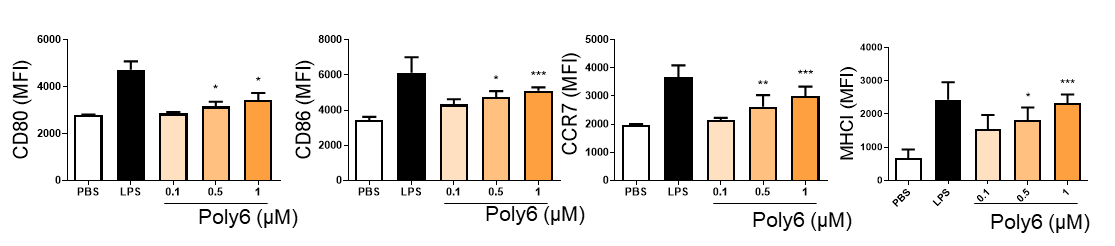** |
| --- |

**Supplementary Figure 3. Poly6 promoted the maturation and migration** **capacity of DCs** Surface expression of co-stimulatory molecules and maturation markers by BMDCs upon exposure to Poly6

| **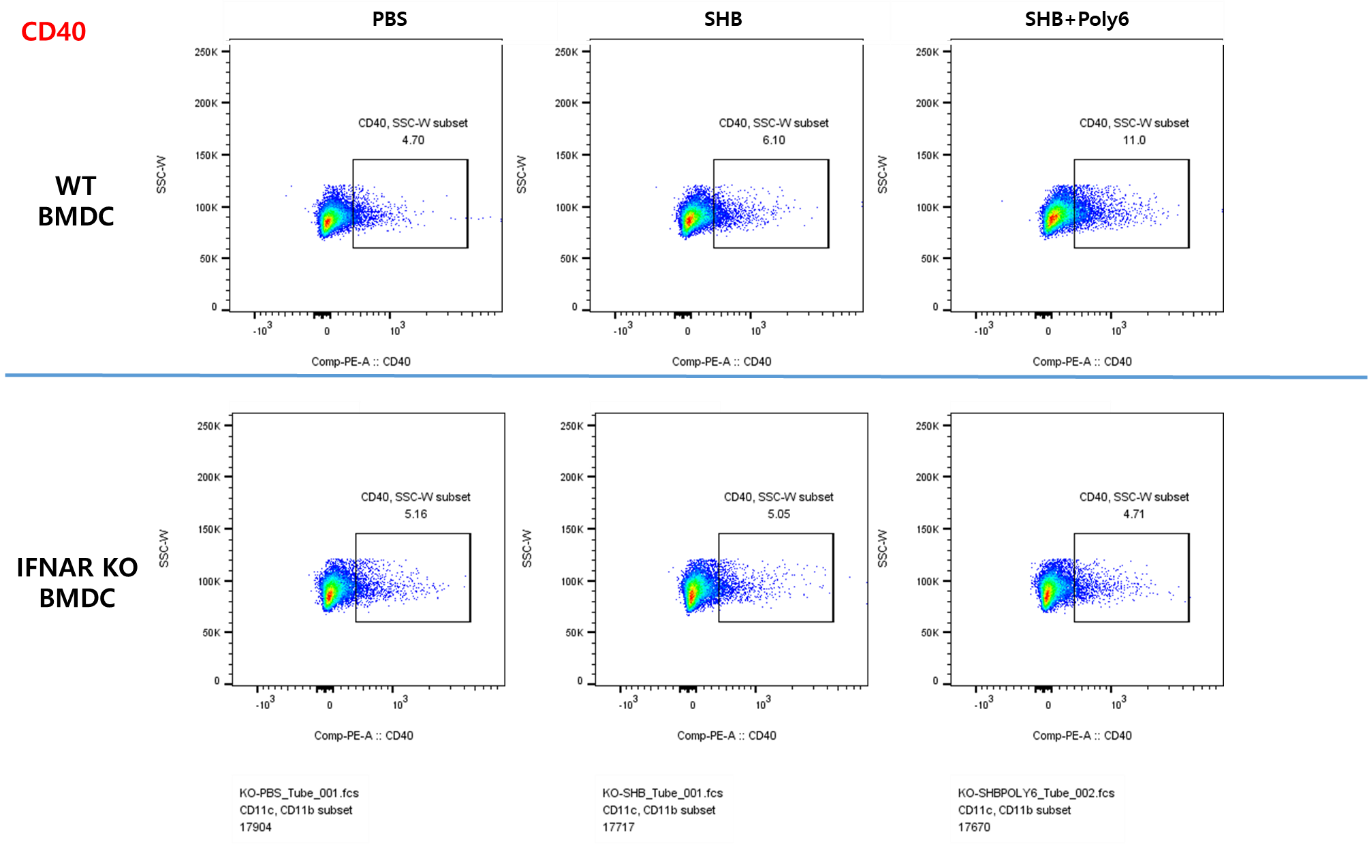** |
| --- |
| **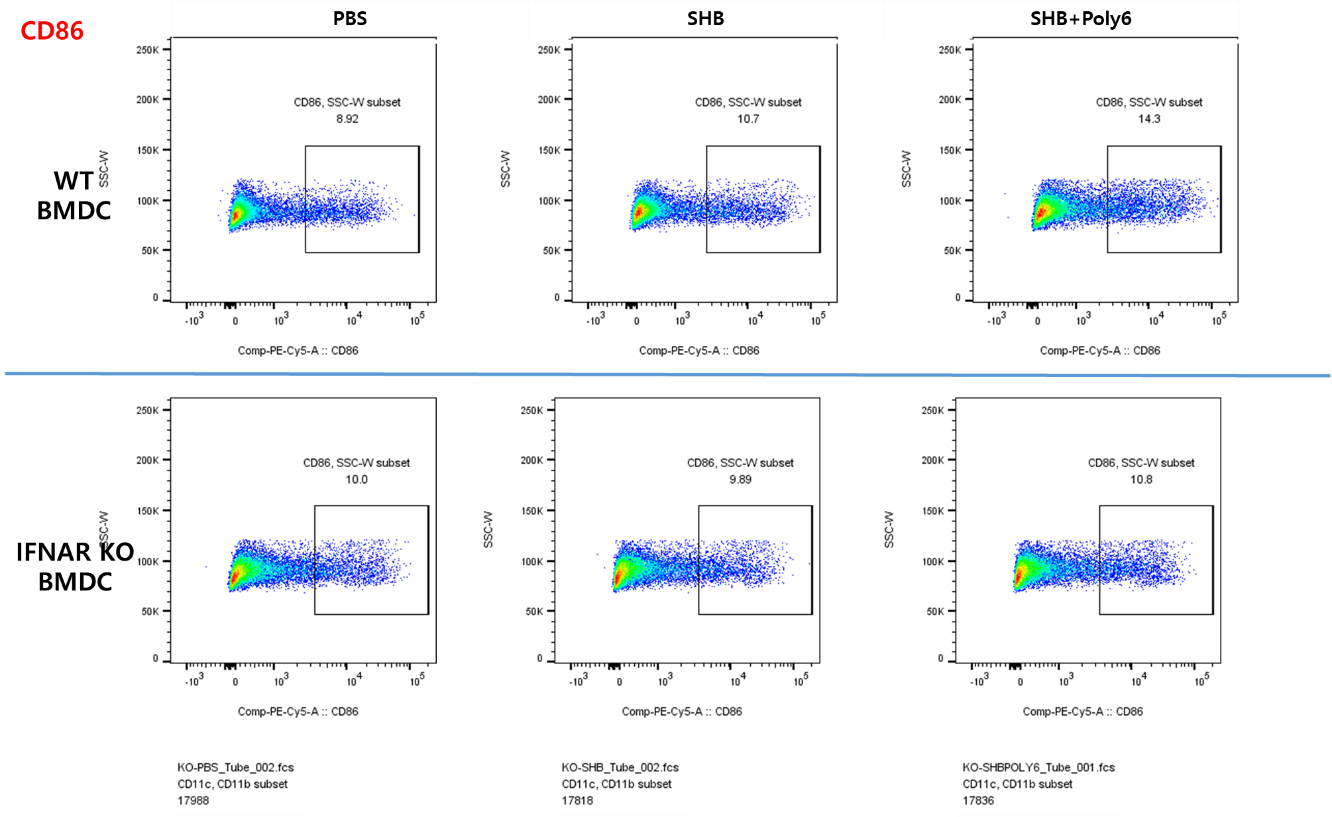** |
| **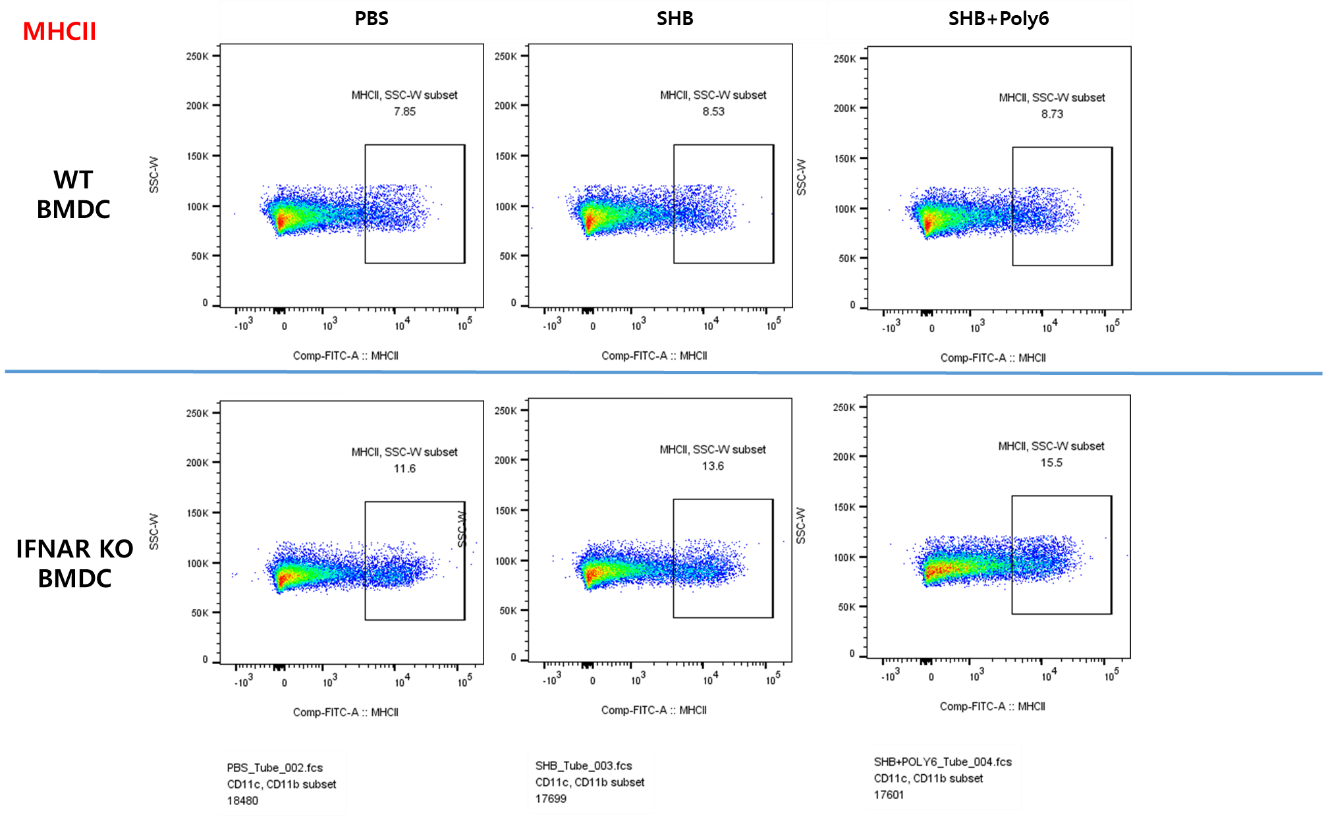** |
| **Supplementary Figure 4. Vaccination with Poly6 in combination with HBsAg induced DC maturation in a type I IFN-dependent manner** Surface expression of co-stimulatory molecules and maturation markers by BMDCs upon exposure to Poly6 in WT or IFNAR KO BMDC |

| 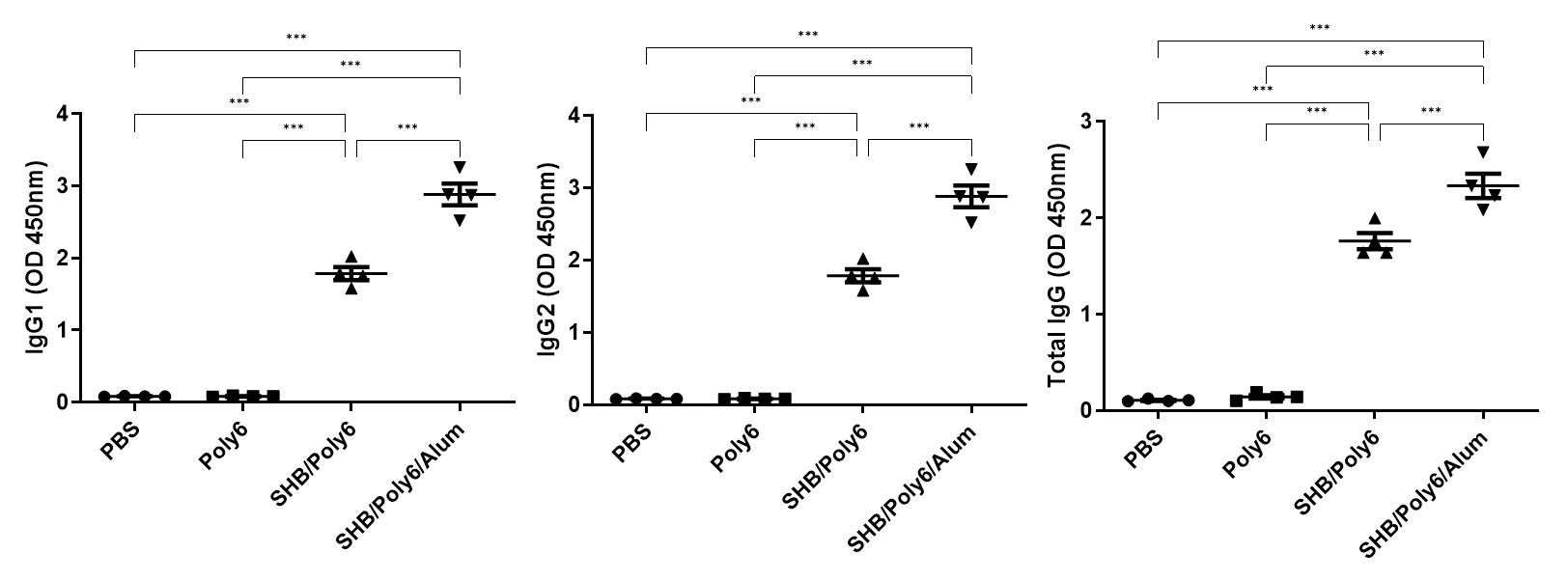 |
| --- |
| 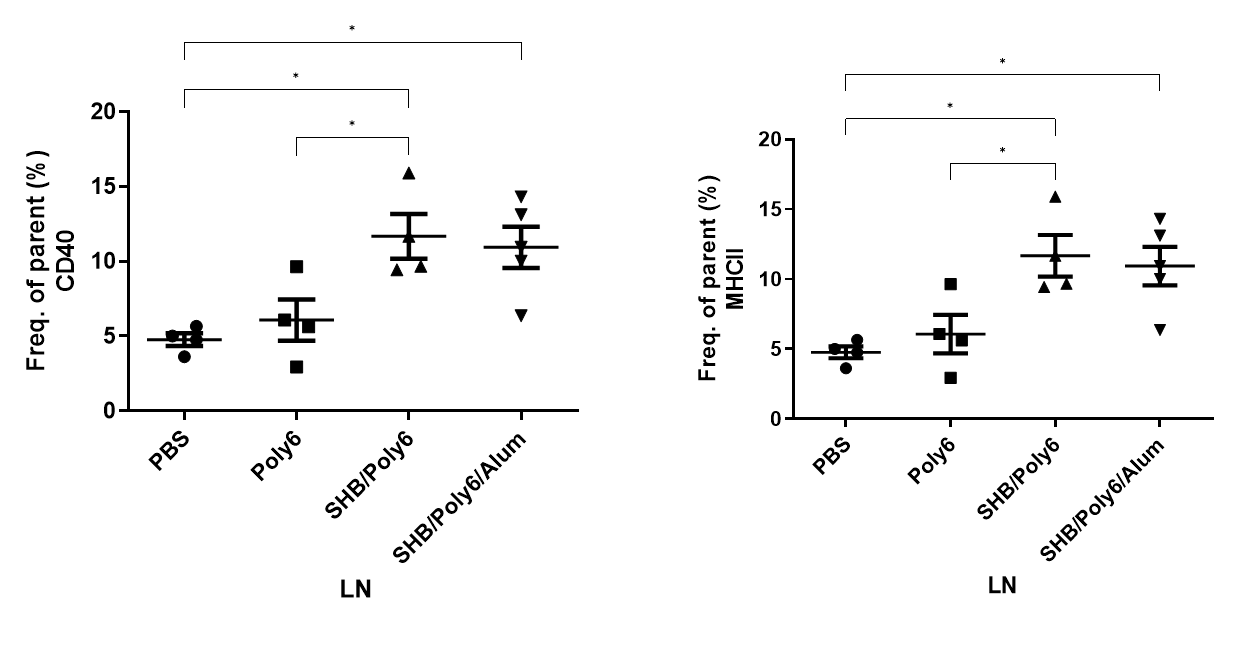 |
| 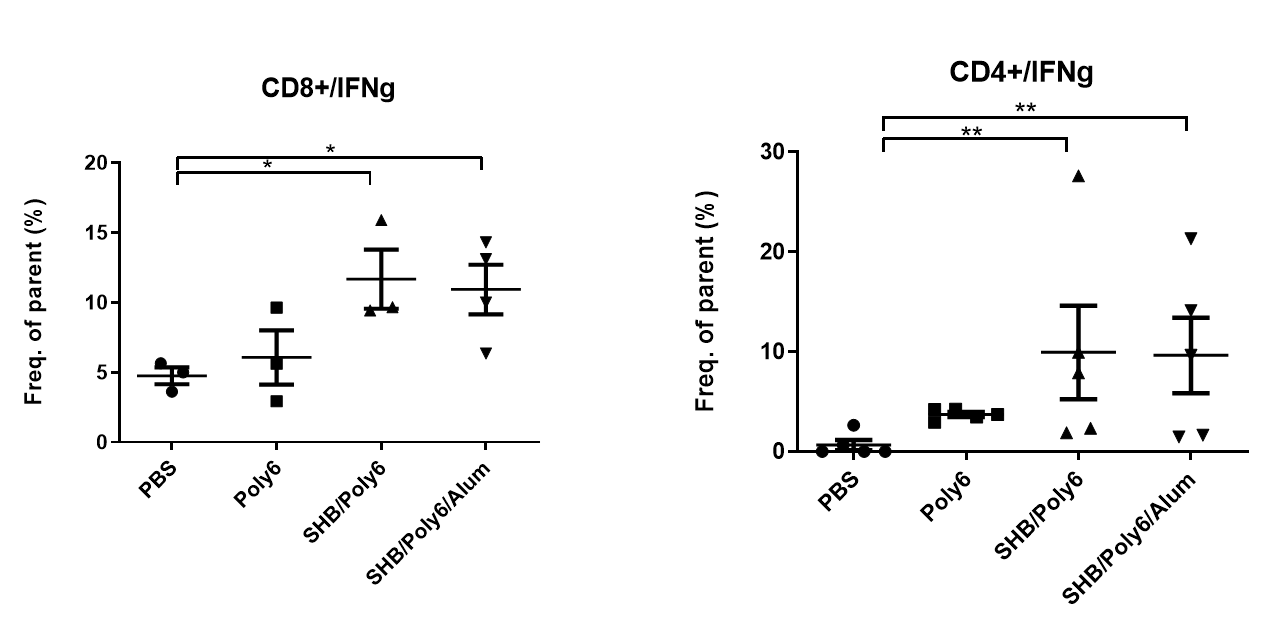 |

**Supplementary Figure 5. Poly6 addition into the HBsAg vaccine protocol potentiated HBsAg-specific cellular and humoral immune responses in C57BL/6 mice.**

|  |
| --- |
| **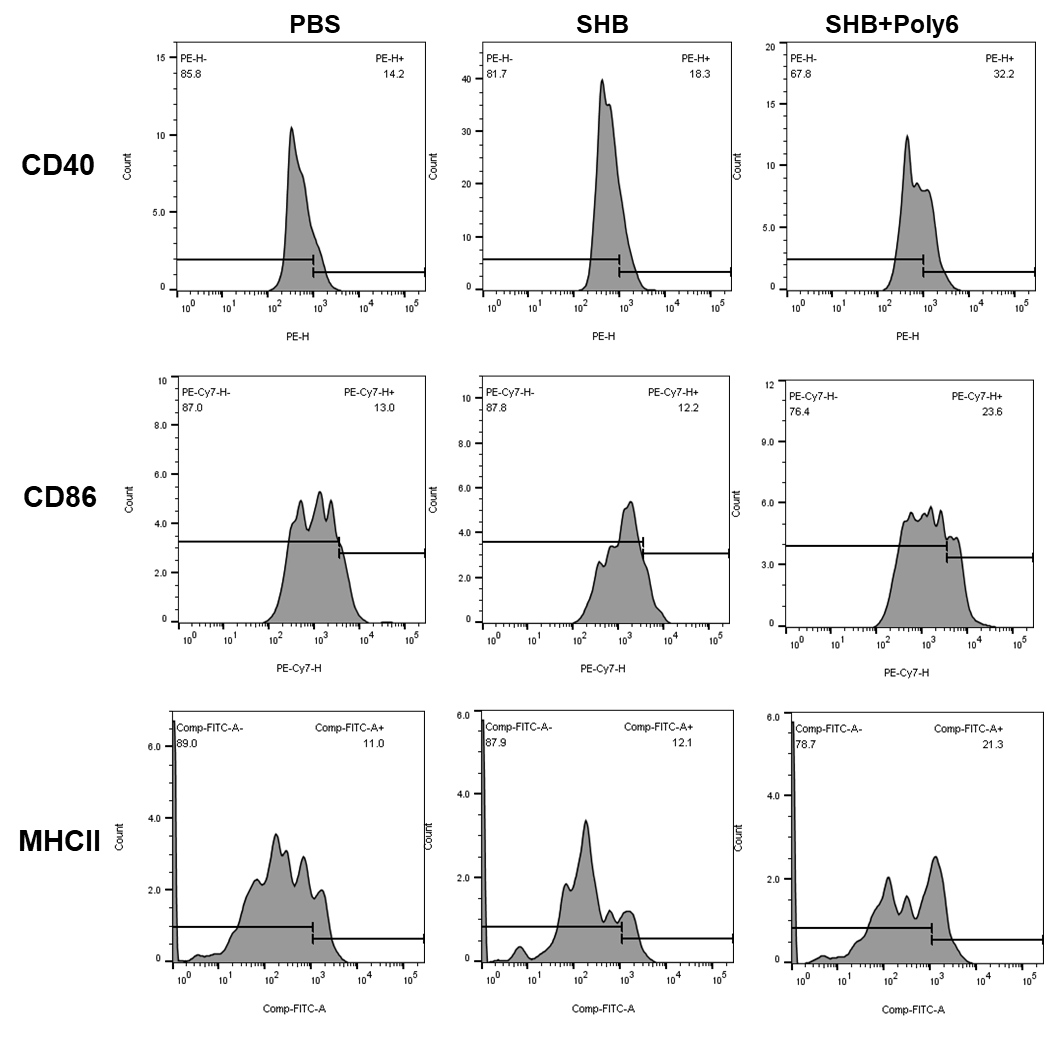** |

**Supplementary Figure 6. Poly6-combined HBsAg vaccination promoted DC maturation in the TG mice** Surface expression of DC maturation markers, CD40, CD86, MHC II, by splenic DCs

| 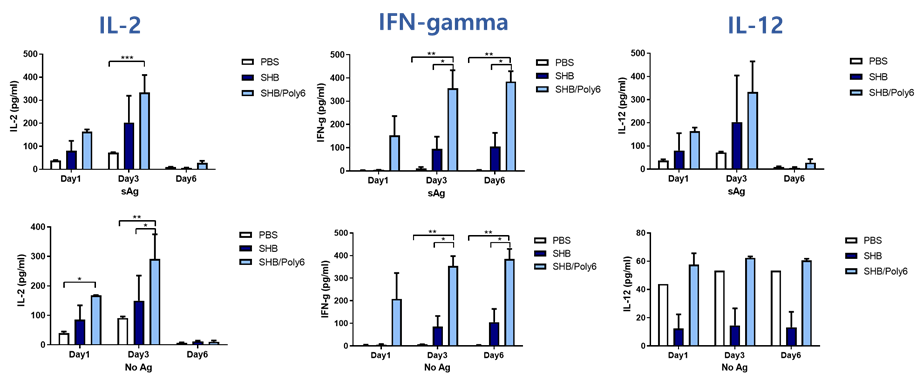 |
| --- |
| 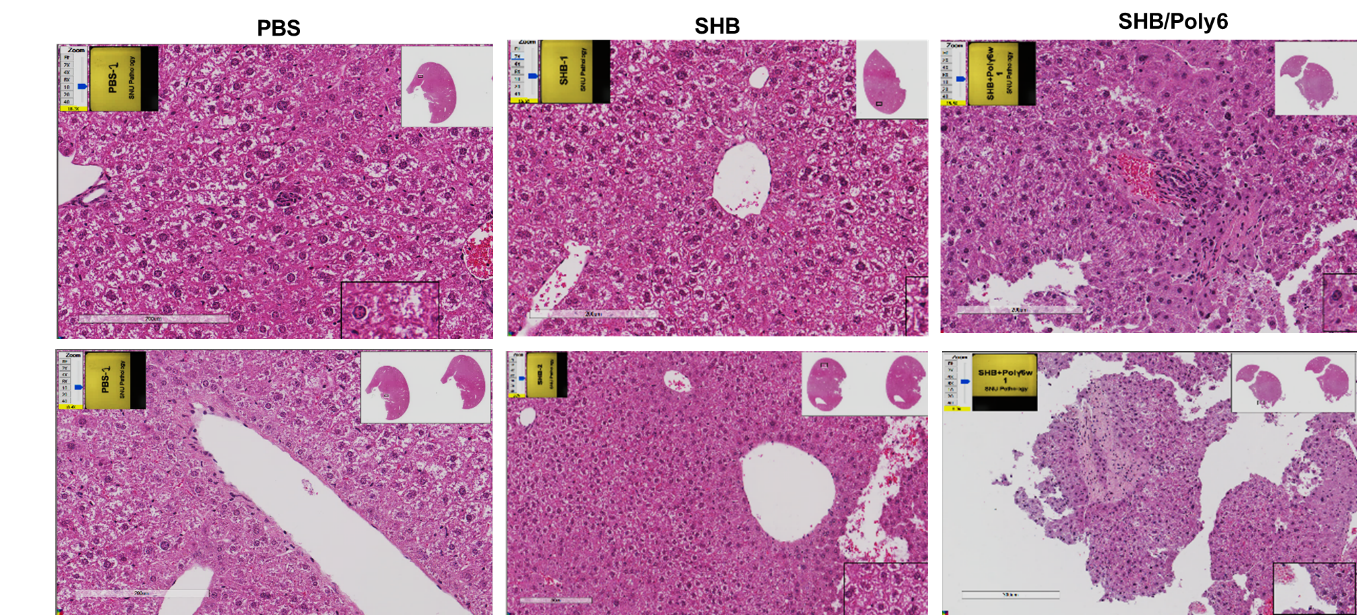 |

**Supplementary Figure 7. Poly6-combined vaccination induced a potent stimulation of Th1 cytokines in the TG mice** Cytokine release in splenocyte-culture supernatant upon in vitro stimulation with or without HBV SHB antigen. IFNγ, IL-2, IL-12 were examined by ELISA. Histopathology of HBV TG mouse livers. Liver tissues were obtained, fixed, sectioned, and stained with H&E. Bar = 200 µm. The results were evaluated for statistical significance by one-way ANOVA with Tukey’s post hoc test. Differences were considered significant when *p < 0.05, **p < 0.01, and ***p < 0.001.

| 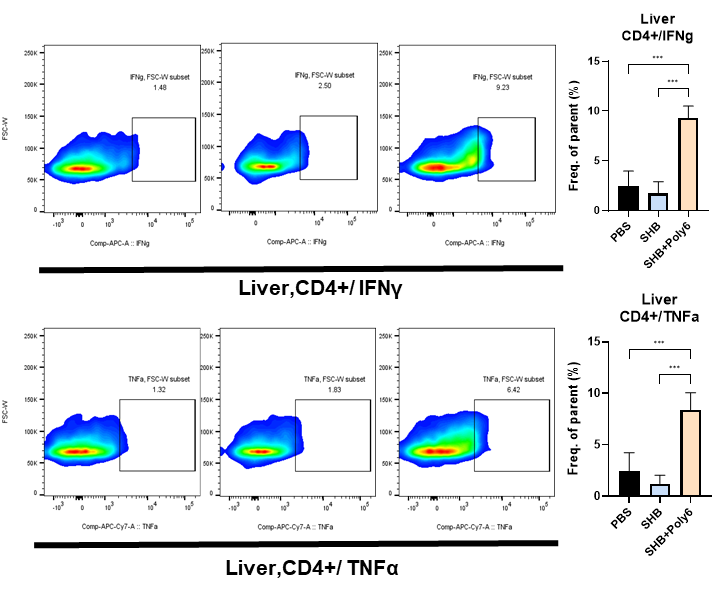 |
| --- |

**Supplementary Figure 8.** Poly6/HBsAg vaccination protocol could induce HBV-specific CD4+ T cells secreting cytokines such as IFN-γ and TNFα in TG mice liver


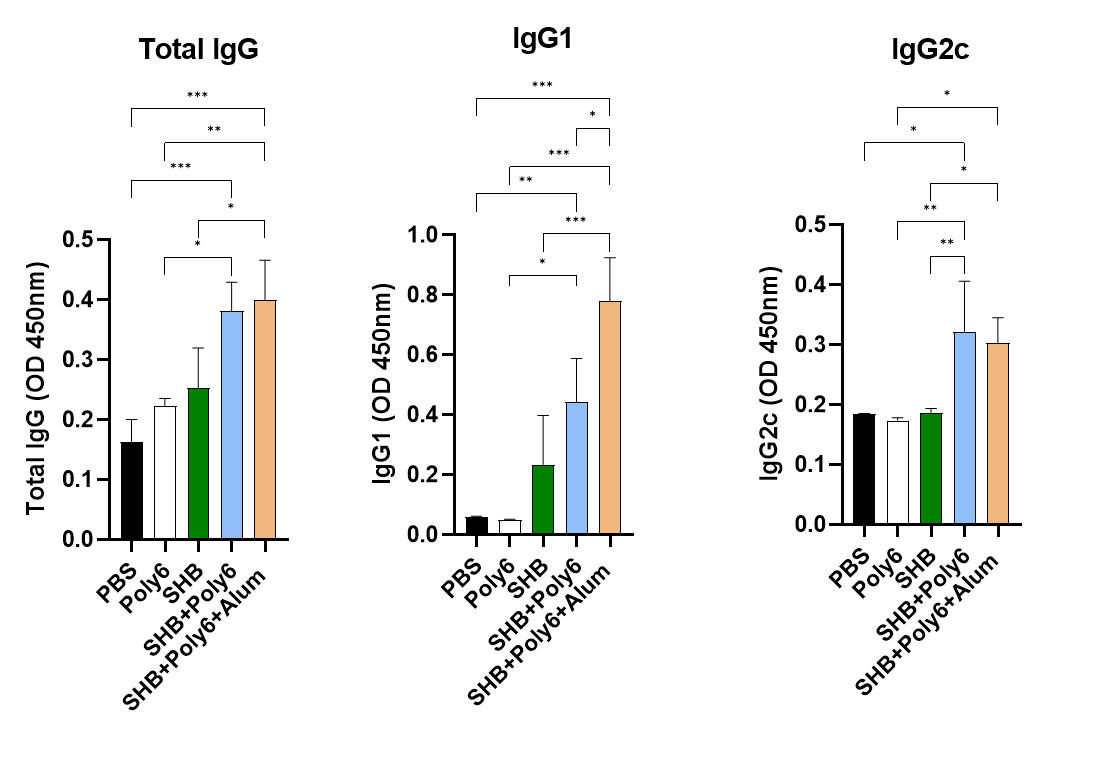


**Supplementary Figure 9. Immunization with poly6-combined HBsAg produced virus-neutralizing antibodies in TG mice** Serum anti-HBsAg total IgG, IgG1, IgG2c were analyzed at week 3 after first immunization.

| 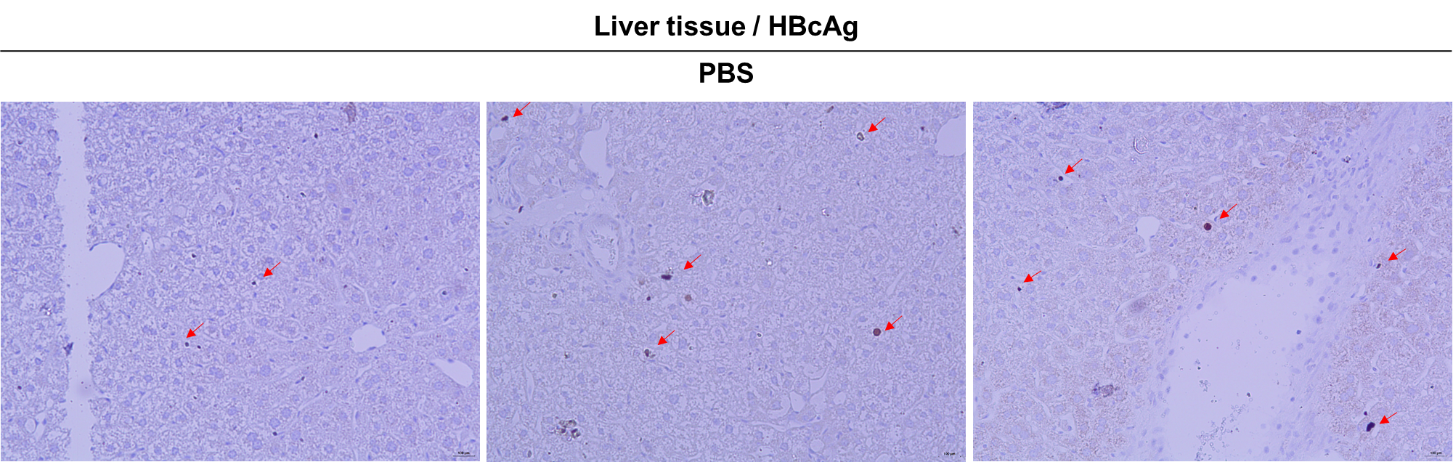 |
| --- |
| 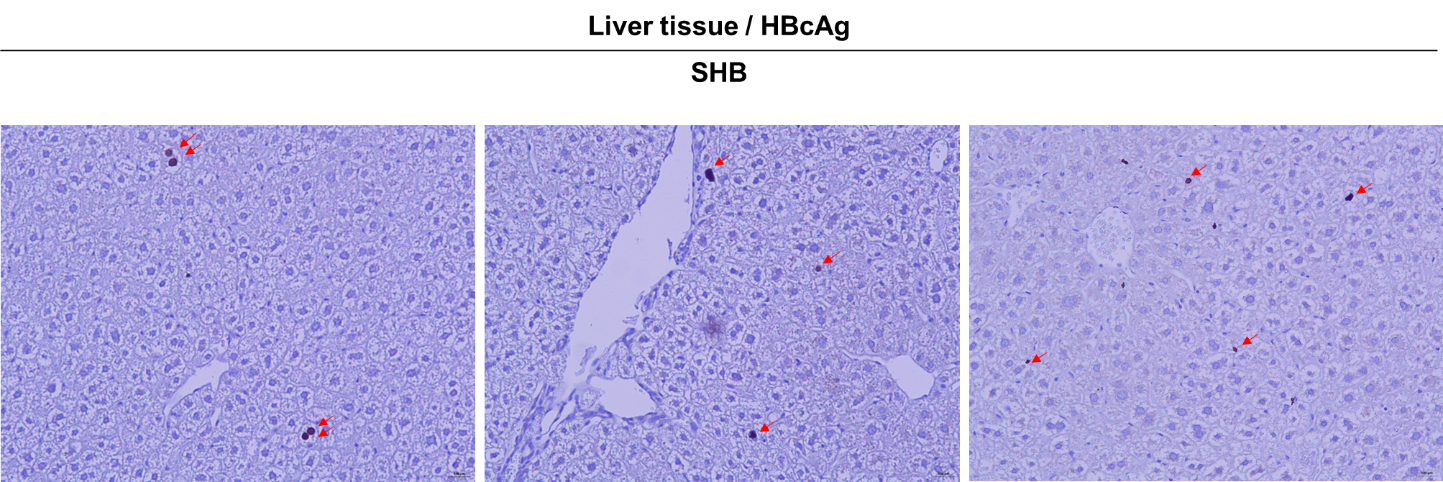 |
| 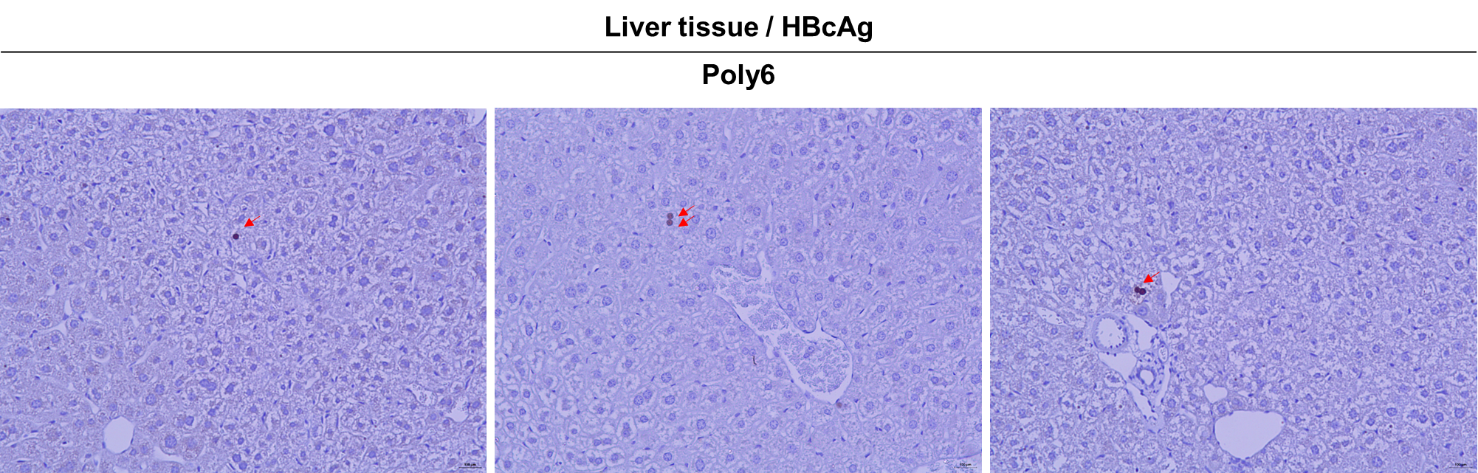 |
| 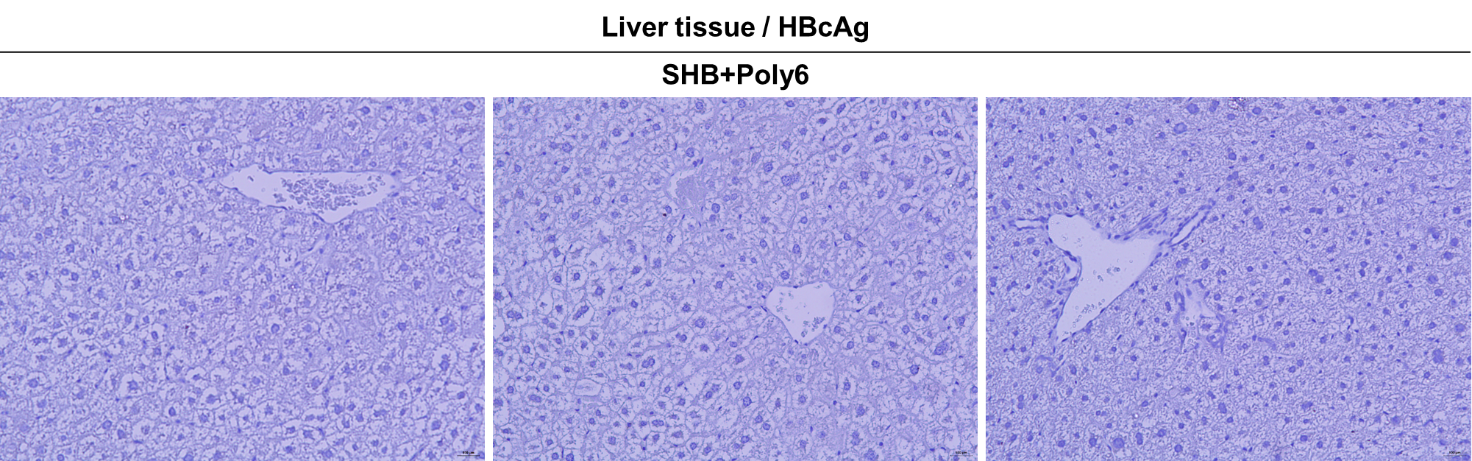 |
| 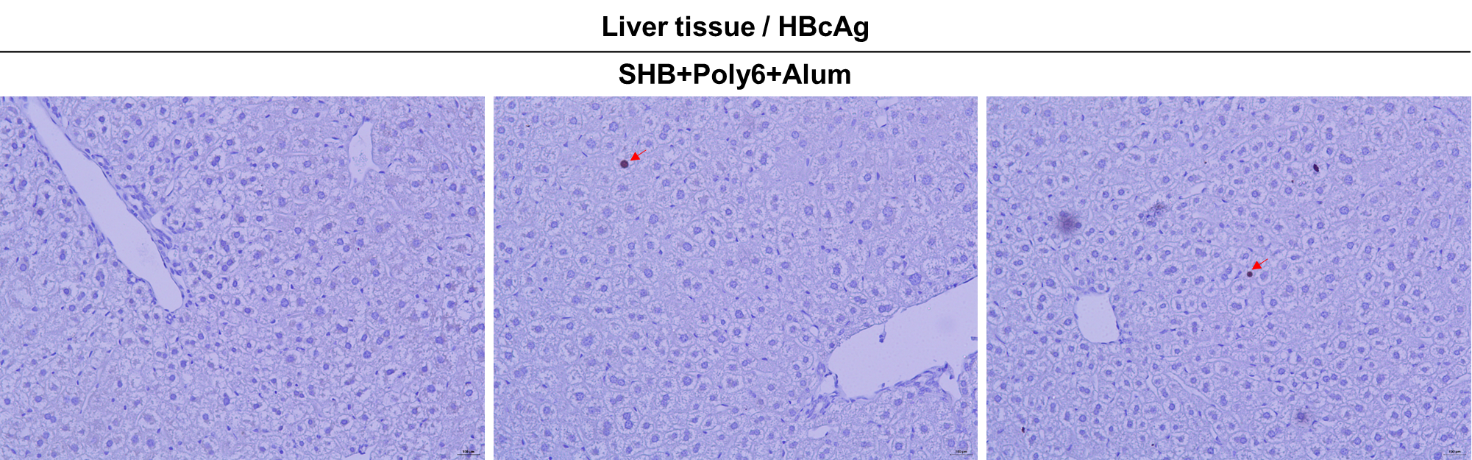 |

**Supplementary Figure 10.** Immunohistochemical (IHC) analysis of HBcAg (arrow) *in vivo*. Liver tissue from the transgenic mice was stained for HBcAg.

| 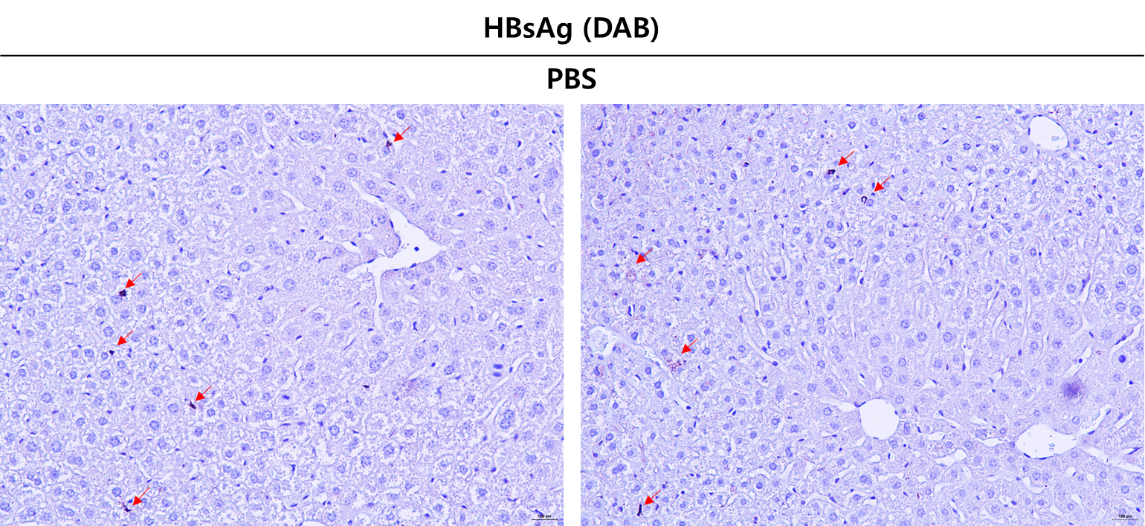 |
| --- |
| 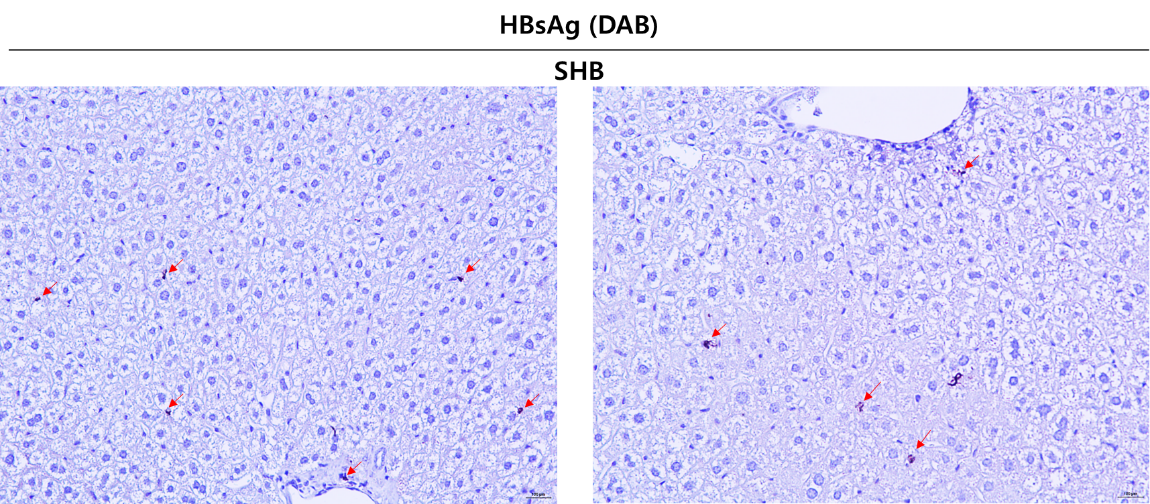 |
| 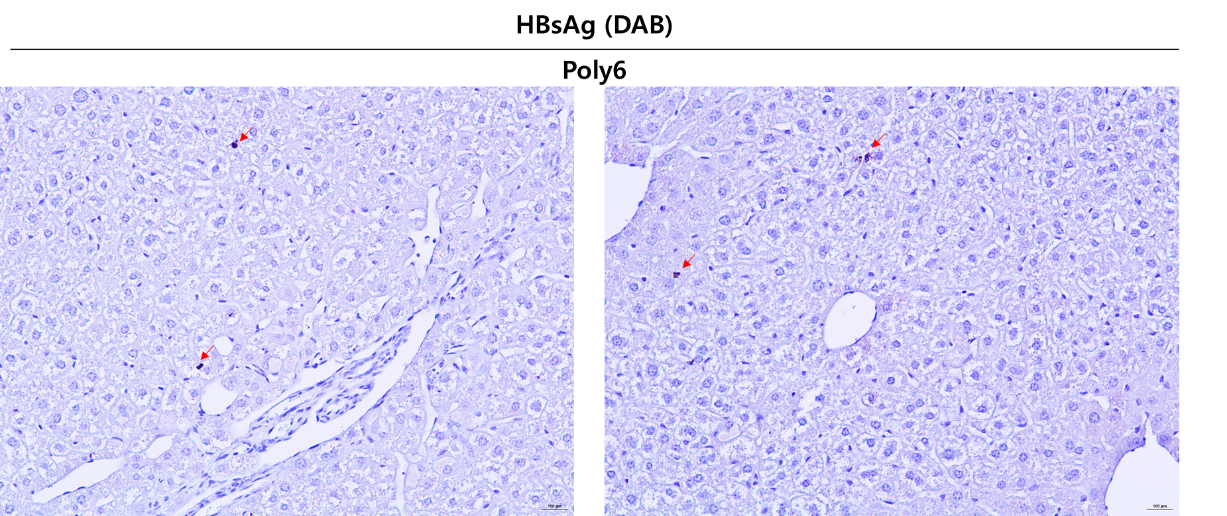 |
| 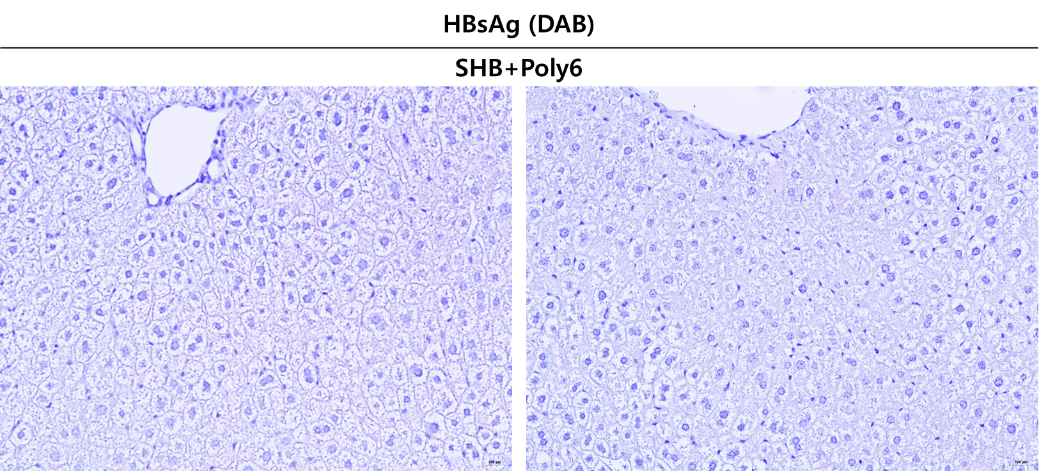 |
| 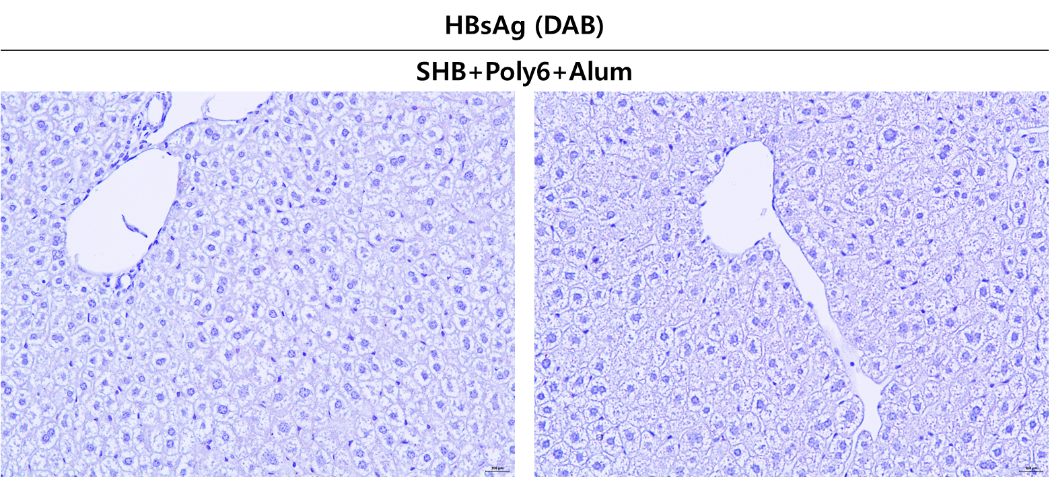 |

**Supplementary Figure 11.** Immunohistochemical (IHC) analysis of HBsAg (arrow) *in vivo*. Liver tissue from the transgenic mice was stained for HBsAg.

**Supplementary Table 1. Safety assessment of Poly6 in ICR mice and necropsy incidence results**

G1

0 mg/kg

Term

G4

20 mg/kg

Term

G3

10 mg/kg

Term

G2

5 mg/kg

Term

Summary: Incidence

Number of Animals:

5

5

5

5

**ADRENAL GLAND**

5

5

5

5

Normal

**AORTA**

5

5

5

5

Normal

**BONE MARROW, STERNUM**

5

5

5

5

Normal

**BRAIN**

5

5

5

5

Normal

**COAGULATING GLAND**

5

5

5

5

Normal

**EPIDIDYMIS**

5

5

5

5

Normal

**ESOPHAGUS**

5

5

5

5

Normal

**EYE**

5

5

5

5

Normal

**GALL BLADDER**

5

5

5

5

Normal

**HARDERIAN GLAND**

5

5

5

5

Normal

**HEART**

5

5

5

5

Normal

**INJECTION SITE**

5

5

5

5

Submitted

5

5

5

5

Normal

**CECUM**

5

5

5

5

Normal

**COLON**

5

5

5

5

Normal

**DUODENUM**

5

5

5

5

Normal

**ILEUM**

5

5

5

5

Normal

**JEJUNUM**

5

5

5

5

Normal

G1

0 mg/kg

Term

G4

20 mg/kg

Term

G3

10 mg/kg

Term

G2

5 mg/kg

Term

Summary: Incidence

Number of Animals:

5

5

5

5

**RECTUM**

5

5

5

5

Normal

**KIDNEY**

5

5

5

5

Normal

**LIVER**

5

5

5

5

Normal

**LUNG**

5

5

5

5

Normal

**LYMPH NODE, MESENTERIC**

5

5

5

5

Normal

**LYMPH NODE, MANDIBULAR**

5

5

5

5

Normal

**SKELETAL MUSCLE**

5

5

5

5

Normal

**NERVE, OPTIC**

5

5

5

5

Normal

**NERVE, PERIPHERAL**

5

5

5

5

Normal

**PANCREAS**

5

5

5

5

Normal

**PITUITARY GLAND**

5

5

5

5

Normal

**PROSTATE GLAND**

5

5

5

5

Normal

**SALIVARY GLAND**

5

5

5

5

Normal

**SEMINAL VESICLE**

5

5

5

5

Normal

**SKIN**

5

5

5

5

Normal

**SPINAL CORD, THORACIC**

5

5

5

5

Normal

**SPLEEN**

5

5

5

5

Normal

**STOMACH**

5

5

5

5

Normal
